# Expression of a multigene mushroom luciferin biosynthesis pathway as a pseudo-polycistron in plants

**DOI:** 10.1038/s41598-025-98717-2

**Published:** 2025-07-14

**Authors:** David Samson, Natalie S. Thompson, Vijay R. Sheri, Sairam V. Rudrabhatla, Wayne R. Curtis

**Affiliations:** 1 Department of Chemical Engineering, The Pennsylvania State University, University Park, PA 16802 USA; 2 Department of Biology, The Pennsylvania State University, Harrisburg, PA 17057 USA; 3 Intercollege Program in Plant Biology, The Pennsylvania State University, University Park, PA 16802 USA

**Keywords:** Plant biotechnology, Gene expression analysis

## Abstract

Mushroom bioluminescence is based on a luciferin/luciferase cycle that includes four catalytic enzymes and a post-translational modifier phosphopantetheinyl-transferase (NpgA). The luciferin cycle includes conversion of the plant cell wall precursor caffeic acid to the mushroom luciferin (3-hydroxyhispidin) substrate—suggesting a logical system for development of in vivo luciferin production rather than addition of exogenous luciferin substrate. *In planta* luciferin biosynthesis is demonstrated from a polycistronic concatenation of the luciferin pathway genes with intervening self-cleaving intein-F2A peptides. Bioluminescence was greater with NpgA transiently expressed separately from the luciferin biosynthesis (LBS) polycistron in *N. benthamiana* but was not detectable in tomato even with all genes on separate promoters. Separation of the bioluminescence reporter and luciferin substrate pathway facilitated studies of mushroom luciferase that reveal instability for the luciferin substrate. Agrobacterium expressing the luciferase is shown to be an effective quantitative biosensor for both the presence of luciferin as well as plant tissue quenching of bioluminescence during tissue disruption. Large plant species-dependent differences in bioluminescence assay quenching are observed, with tomato displaying instantaneous suppression comparable to wild-type negative controls. Although bioluminescence is observed using transient luciferin/luciferase co-expression in tobacco (*N. benthamiana*), luciferin could not be isolated for use in exogenous assay. The challenge of using the mushroom luciferin biosynthesis pathway in transgenic plants as a complementation reporter is discussed in the context of our inability to detect luciferin in tomato transgenic lines after homozygous segregation using digital PCR. The utilization of in vivo mushroom luciferin biosynthesis is anticipated to be increasingly effective in the future based on ongoing gene improvements in pathway biosynthesis subject to the constraint of substrate instability.

## Introduction

**Fig. 1:**
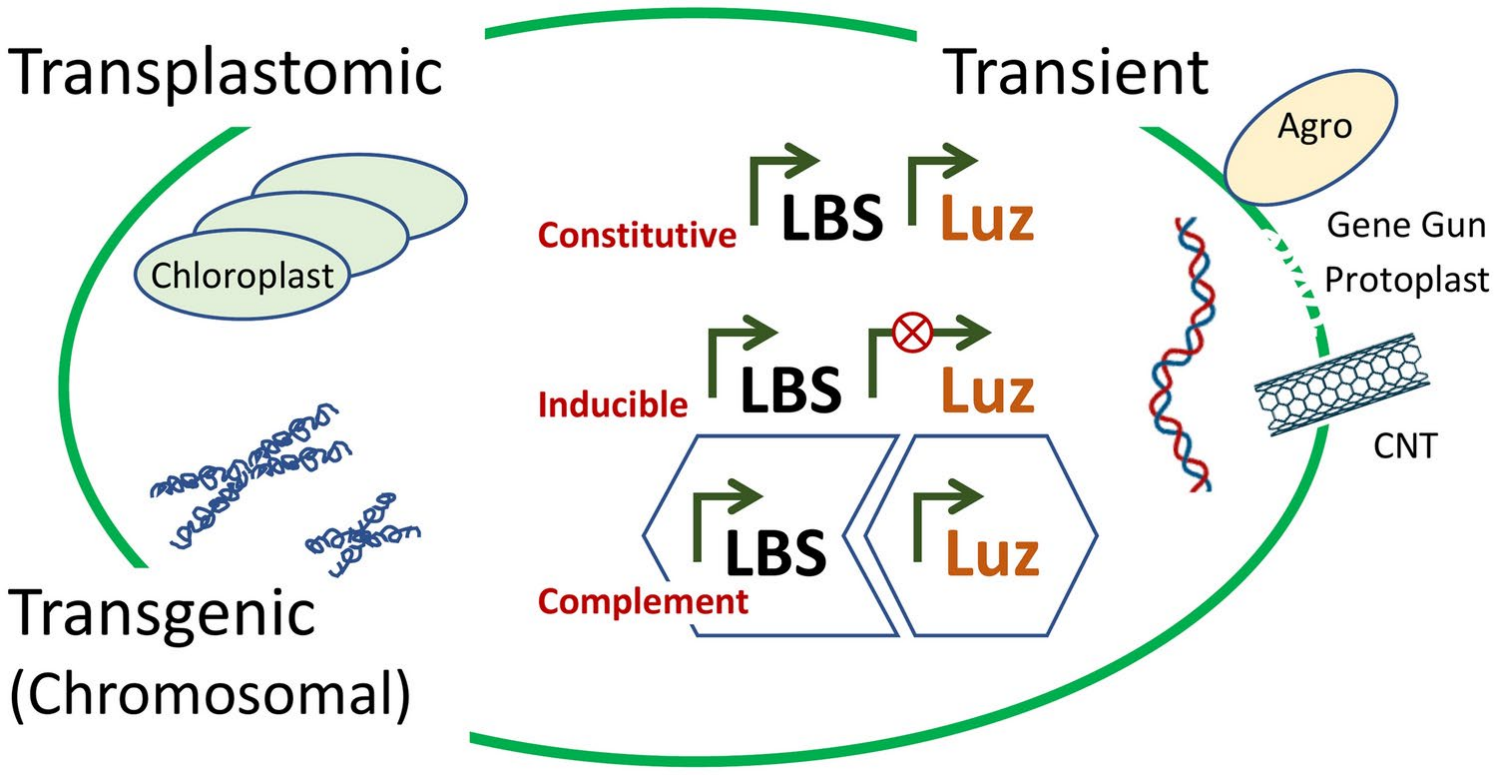
Approaches to introducing the luciferin biosynthesis pathway (LBS) and luciferase (Luz) into plants to create bioluminescence. Introduction of constitutive LBS + Luz has been accomplished by transplastomic transformation of bacterial genes in chloroplasts, transgenic transformation of the chromosome with mushroom genes and transient expression of mushroom genes using Agrobacterium (Agro) or other DNA delivery methods. The use of an inducible promoter driving the luciferase has been tested with transient Agro expression. Complementation in which a transgenic LBS plant generates luciferin substrate for reaction with luciferase expressed by other means (e.g. tissue assay, viral vector, or transient screening of luciferase) has tremendous potential for plant physiology study but has not yet been reported.

Although bioluminescence has independently evolved in bacteria, protists, fungi and insects, it has not been discovered in higher plants^1^. It is also relevant to note the lack of symbiotic bioluminescent relationships in plants that are found in animals, particularly deep-sea marine life^2^. This would suggest that plants have opted for alternative biochemical strategies to improve reproduction. At the same time, plants represent a particularly versatile platform in which to explore transgenic bioluminescence due to the prokaryotic character of chloroplasts, eukaryotic chromosome and the diversity of plant biotechnology methodologies that have been developed to facilitate transient or ectopic gene expression. Since the light-emitting pathway involves a luciferase (Luz), the simplest approach to achieving bioluminescence is the addition of the luciferin—which represents the typical use of luciferase as a reporter gene such as evaluating gene regulation^3,4^.The limitations of this application are the penetration of the luciferin substrate, as well as the associated expense. The focus of this work is the concept of introduction of the biosynthetic pathway for luciferin biosynthesis (LBS) in complementation with the luciferase for bioassay applications. This goal is different from most efforts which involve making ‘glowing plants’ which is not only aesthetic, but useful as a sustainable basis for low-level light production^5,6^. The schematic of Fig. 1 provides a basis for clarifying the status of creating a luciferin producing (LBS +) plant phenotype in the context of the majority of efforts to achieve auto-bioluminescent (LBS-Luz +) phenotype that is prevalent in the popular press and commercial efforts such as Planta, LLC (Moscow) and Light.bio.

The first report of autoluminescent plants involved transformation of the bacterial luciferase and luciferin-D genes for constitutive expression in tobacco chloroplasts^7^. This approach is based on the simplicity of bacterial operons, and the ability of chloroplasts to process multiple genes from a single promoter; however, the creation of transplastomic plants is extremely challenging due to the requirement for converting hundreds of chloroplasts to achieve a homoplastomic plant^8^. The more recent, and brighter autofluorescence has been achieved with the constitutive expression of mushroom LBS + Luz that is biochemically compatible with plant metabolism due to the luciferin pathway sharing an intermediate with plant cell wall biosynthesis^6^. There are substantial ongoing efforts to improve these enzymes; however, these improvements have focused primarily on animal and bacterial systems—most likely due to the simplicity of high through-put laboratory evolution in these systems^9^. The generation of plant transgenics by any methodology is comparatively inefficient—requiring years of selection and segregation.

While major advances in transgenic plant regeneration are being made using transient expression of morphogenic transcription factors to achieve embryogenesis or meristem formation^10–12^, this commitment to producing genetically transformed offspring requires extensive periods of time (years) that are not conducive for most academic research. This has led to the extensive use of transient ectopic transformation where the DNA is delivered by either the plant pathogen *Agrobacterium tumefaciens*^13^, transfection of protoplasts^14^, biolostic ‘gene gun’ technology^15^, and nano-particle delivery^16^. The use of the Agrobacterium delivery system introduces the concerns for bacterial based expression, which have been overcome through the insertion of an intron in both the *Renilla* luciferase^17^, as well as the *Neonothopanus nambi* mushroom luciferase. The speed and versatility of transient gene expression has facilitated LBS + Luz expression, including an examination of inducible and circadian promoter function using the mushroom luciferase system^18^.

The use of plant viral vectors is of particular utility since it provides for self-replicating transgenes that can amplify and move within plants^19^. Many viral vectors utilize other transient expression methods to launch the virus and augment gene expression. The scalability of viral vector delivery of DNA was the motivation behind our research to provide ‘gene therapy’ to plants in the field through the delivery and systematic movement in plants. It is in this context, that we distinguish our goal of a complementary LBS + Luz system through the generation of transgenic plants that produce the luciferin in the absence of the luciferase. Only through complementation of the transgenic by other means (e.g. transient or viral vector) is bioluminescence observed.

**Fig. 2:**
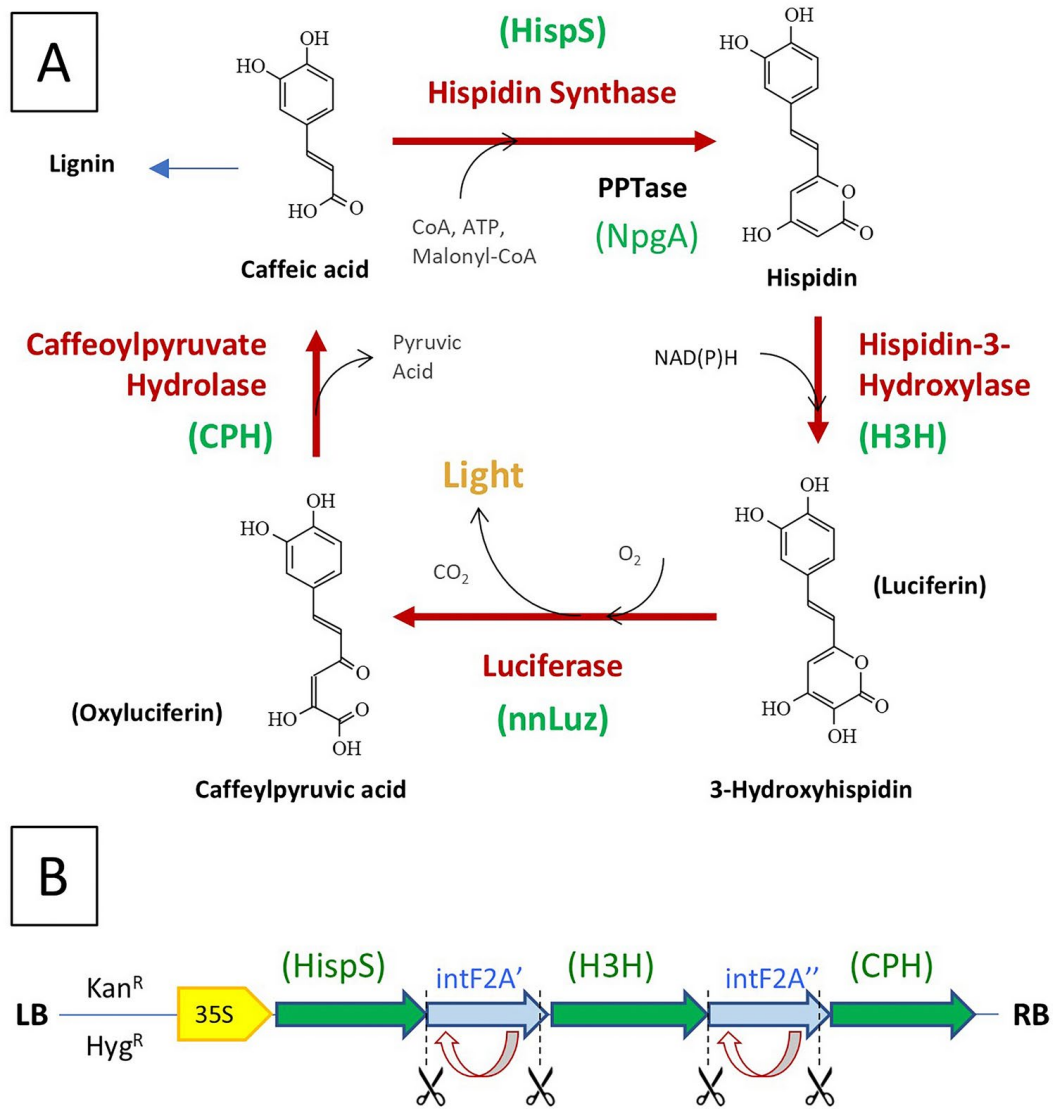
Luciferin biosynthesis pathway and a polycistronic expression construct. (**A**) Mushroom bioluminescence cycle noting its relation to cell wall lignin biosynthesis. (**B**) Example pseudo-polycistronic LBS construct where the three biosynthesis and recycle genes are expressed as a single mRNA followed by ribosome skipping which functionally ‘cleaves’ at the 3’ of the intF2A amino acid sequence followed by intein self-cleavage at the 5’. The use of non-redundant intein linker coding sequences was chosen to reduce potentially unstable nucleotide tandem repeats.

The mushroom luciferase biosynthesis pathway was chosen for its potential for generating endogenous luciferin substrate. The metabolic cycle that produces fungal bioluminescence in *N. nambi* starts from caffeic acid (trans-caffeate; Fig. 2), which has two malonate groups added in the ATP-dependent hispidin synthase (HispS) reaction. The resulting molecule, hispidin, is hydroxylated to produce 3-hydroxyhispidin (the fungal luciferin) in the NADPH-dependent and oxygen-dependent Hispidin-3-Hydroxylase (H3H) catalyzed reaction. Luciferin is then reacted with oxygen to produce light in the luciferase (nnLuz) catalyzed reaction. The resulting molecule, caffeylpyruvic acid (the oxyluciferin), is hydrolyzed by caffeylpyruvate hydrolase (CPH), to produce pyruvate and caffeic acid (the starting molecule). In addition to the specific enzymes of the catalytic cycle, HispS requires post-translational modification (a phosphopantetheinylation) to one of its domains for activity. This modification for the transgenic pathway is provided by the *Aspergillus nidulans* 4′-phosphopantetheinyl transferase NpgA gene. While the pathway is biochemically compatible with plant metabolism, and utilizes cell wall biosynthesis precursors, the introduction of the mushroom luciferin and biosynthesis pathway (without the luciferase reporter) requires 3–4 genes, with the largest gene, nnHispS, being 5094 bp. Combining this with plant promoters that are also 1–2 kb in size, the construct for introduction into the plant is on the order of 20 kb). Successful production of luciferin then requires coordinated expression of all these transgenes.

To reduce the size of the transgene construct, we sought to create a pseudo-polycistronic message that would encompass the LBS by generating a single transcript with the proteins being separated by self-cleaving intein-F2A hybrids (Fig. 1B) as described for the simultaneous expression of multiple reporters^20^. The ‘ribosome skipping’ function of the viral F2A protein, combined with the intein self-cleavage provides for both 5’ and 3’ removal of the linker from the polyprotein segments^21^. In this manner, there is a requirement for only one promoter, and with the size of the intein-F2As being 675-bp, a reduction of construct size to 11-kb is readily possible. This pseudo-polycistron has reduced flexibility in terms of tuning levels of expression of individual genes but does provide for comparable levels of expression that are localized within the same cells.

We describe here the successful generation of several forms of the luciferin biosynthesis pathway separated from its cognate mushroom luciferase reporter. By generating a transient expression vector based on an intron-containing luciferase, a successful in vivo expression of the entire luciferin biosynthesis cycle as two complementary transcripts is demonstrated. Efforts to produce transgenic tomato with functional luciferin biosynthesis achieved some indications of success for initial T0 transformation, but luciferin expression and/or transient expression of the luciferin were not observed in segregated transgenic tomato plants. A key constraint for the use of this bioluminescent reporter, is the instability of the substrate and the extremely rapid quenching of the bioluminescent assay in the presence of disrupted plant tissues. The opportunity and constraint of using the mushroom bioluminescent system as a reporter is discussed in the context of the improved understanding of its observed kinetics, including the use of the nnLuz enzyme expressed in bacteria as a biosensor for successful luciferin biosynthesis.

## Results

A series of luciferin biosynthesis (LBS) pathway genes and the enzyme-modifying phosphopantetheinyl-transferase were constructed as listed in Table 1. Luciferase was excluded from these constructs so that it could be independently introduced as a complementation reporter. The pLSU2 binary vector contains kanamycin plant selection (nptII) and the pLSU4 binary vector contains hygromycin selection (HPH) for the generation of transgenic plants in addition to transient expression. The cloning was confirmed by sets of PCR primers to provide sequencing of overlapping segments—for example Ly117 sequencing involved 10 primer sets (see Supplemental File 2, S2-1A). The individual pathway components were also generated as analogous individual expression constructs under the control of the P35S promoter and TMV Ω’ 5’ UTR for enhanced translation. More details of constructs are provided in Materials and Supplemental File 2.

**Table 1:** Polycistronic constructs for various permutations of LBS pathway components and the complete LBS polycistron to scale.

P35S[TMV Ω’]:H3H:oIntF2A:CPH:Tnos	Ly115 in pLSU4
P35S[TMV Ω’]:HispS:nrIntF2A-1:H3H:oIntF2A:CPH:Tnos	Ly117 in pLSU4
P35S[TMV Ω’]:NpgA:nrIntF2A-2:HispS:nrIntF2A-1:H3H:oIntF2A:CPH:Tnos	Ly118 in pLSU2


Each construct was placed in the pLSU binary vector under the control of a 0.4-kb cauliflower mosaic virus constitutive promoter (P35S), the 5’ untranslated region (5’ UTR) of tobacco mosaic virus (TMV) and a nopaline synthase terminator (Tnos) between the left and right borders (LB, RB) of the T-DNA. Non-redundant intein-F2A linkers separate the LBS genes (see Supplemental S2-3 for intein sequences). Ly115 / Ly117 / Ly118 are the transgenic tomato lines generated with the associated constructs.

**Fig. 3:**
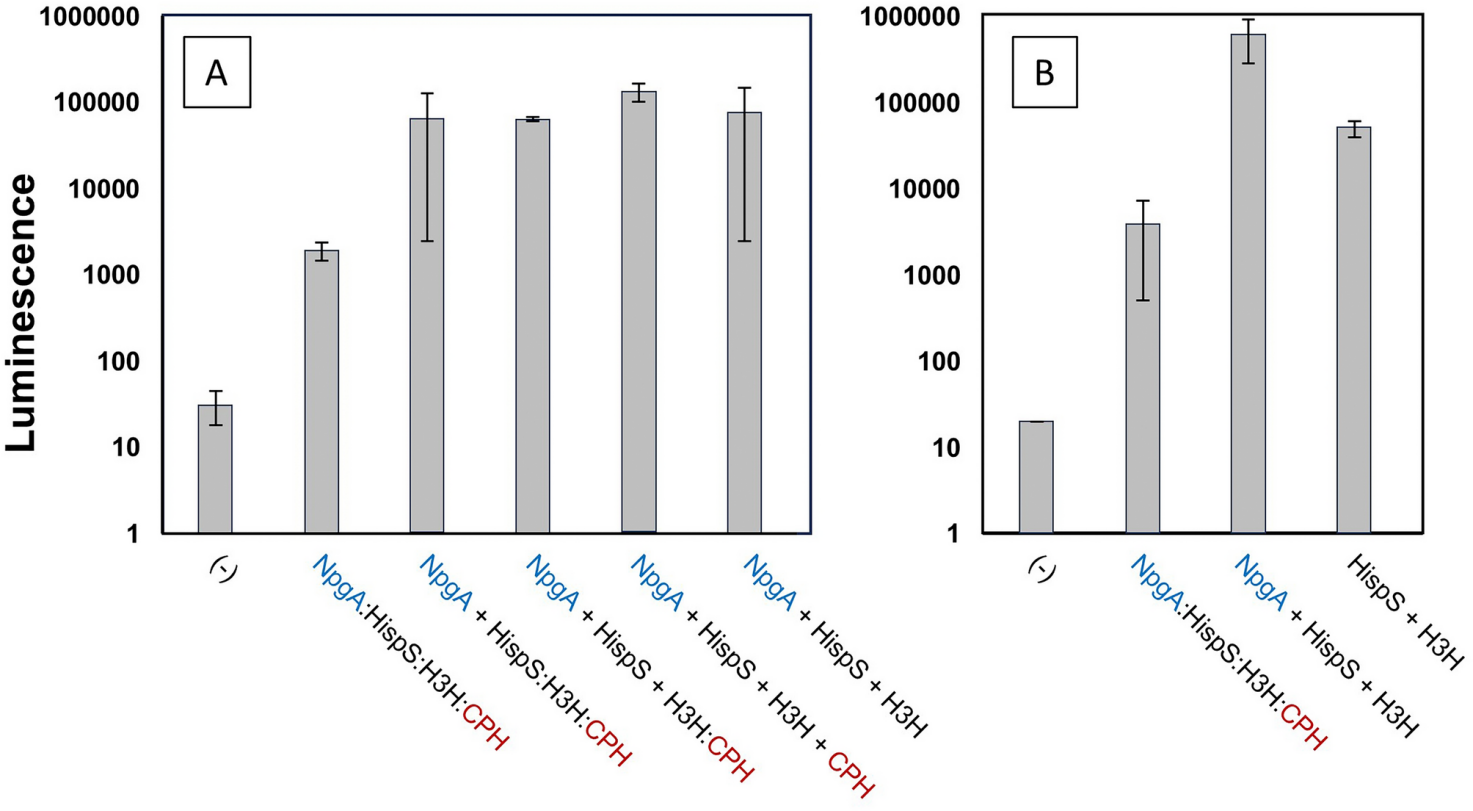
Transient expression of fungal luciferin biosynthesis transformation constructs along with a fungal luciferase construct in 5-week-old *N. benthamiana* plants. (**A**) Leaf disc luminescence was measured in duplicate from independent tissue samples for 10 s in a Promega GloMax 96 luminometer. Luciferin biosynthesis genes separated by plus signs indicate separate T-DNAs, and colons indicate IntF2A polyprotein linkers. Specific linkers used articulated in Supplementary File 2. (**B**) Leaf disc luminescence was measured in triplicate focusing on the requirement for posttranslational modification by NpgA. Error bars are standard deviations.

The functionality of these constructs was confirmed using transient expression of the LBS pathway components co-expressed with the nnLuz fungal luciferase in *N. benthamiana* as shown in Fig. 3A. Expression at 3 days post infiltration (dpi) was an order of magnitude higher than 4-dpi, where the dynamic response for this simple leaf disk bioluminescence reading went through a distinct maximum after 30 min (Supplemental File 1, Figure S1). The full polycistronic binary vector construct (pLSU2//NpgA:HispS:H3H:CPH) displayed bioluminescence that was considerably lower than the constructs that included the phosphopantetheinyl-transferase (pLSU4//NpgA) separately. The permutations of the separate HispS, H3H and CPH are comparable. Surprisingly, the pathway combination that omitted the CPH enzyme which recycles reacted luciferin (caffeoyl-pyruvate) was comparable to the analogous combinations that included this enzyme. This suggests that for the conditions of transient expression, the formation of luciferin is not limited by substrate recycle. An additional experiment was conducted to assess the requirement for NpgA for the HispS and H3H transiently co-expressed as individual T-DNAs (Fig. 3B). The treatment without NpgA displayed lower bioluminescence than the treatment with NpgA, although the expression was still quite high suggesting that the heterologous NpgA improved HispS activity, but was not an absolute requirement. The results are also suggestive of higher absolute levels of enzymes expressed from separate promoters, as well as corroborating a minimal requirement for recycle of caffeic acid.

Numerous observations of transient expression are worth noting towards the development of this bioluminescence technology. *N. benthamania* leaf tissue which displayed quantifiable luminescence in luminometers was not detected under a Thorlabs CEA 1400 optical bench that was designed for this purpose with an 8051 M-USB 8.0 megapixel CCD camera with an exposure time of 1 min and maximum gain of 500. Transient expression in *Solanum lycopersicum* (tomato) with both the polycistronic gene and co-expression of the four enzymes separately did not display quantifiable luminometer bioluminescence. These results indicate that the levels of light production are quite low. Comparative luminescence measurements for the Light.bio Firefly Petunia^TM^ are provided in Supplemental File 1, Figure S2). We observe that both *E. coli* and Agrobacterium harboring the LBS binary vectors grew significantly slower than strains with unrelated or empty vector binary vectors. As an initial test for possible deleterious mutations that that could result from this negative selective pressure, the Ly118 plasmid was extracted from the *E. coli* strain and subjected to agarose gel electrophoresis (Supplemental File 1, Figure S3). Six bands ranging in size from 1-kb to over 20-kb were observed, which suggested extensive homologous recombination. This necessitated a recovery of the functional plasmid and placement in recombination-deficient (RecA-) strains of *E. coli* and Agrobacterium, NEB Stable and AGL-1, respectively. These bacterial strains were ultimately used to create transgenic plants expressing this gene. A final observation consistent with a negative selective pressure against this construct, was revealed in full plasmid sequencing of the Ly117 from an Agrobacterium culture that displayed recovered growth rate and loss of transient bioluminescence. This plasmid contained a 1324-bp, IS3 family transposase IS426 transposable element that disrupted the polycistronic reading frame in the HispS gene (supplemental File 2, S2-2D). Since full plasmid sequencing requires greater than 50% consensus, this clearly indicates that this disruption provides favorable selection.

**Fig. 4:**
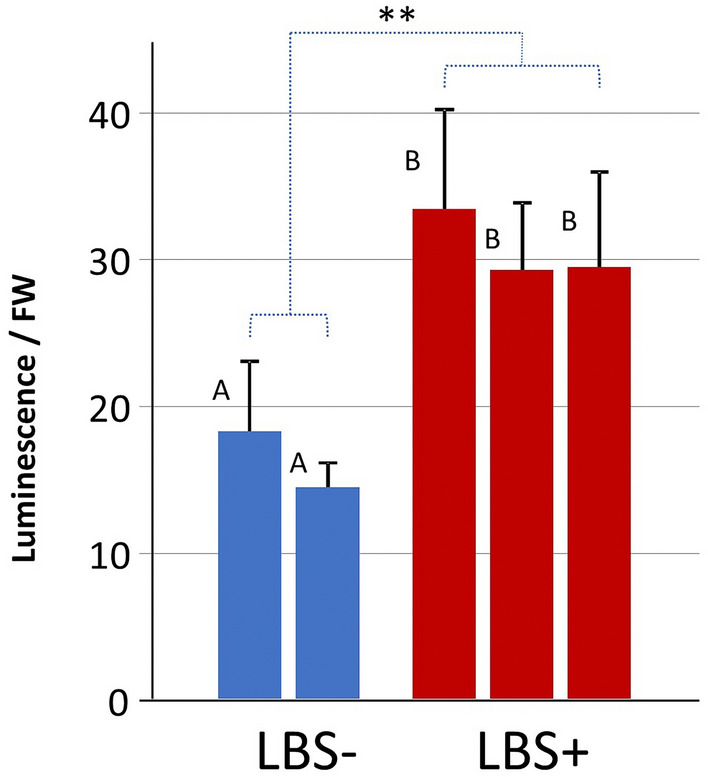
Bioluminescence of leaf extracts of T0 Ly118 regenerated plants in the presence of luciferase extracted from transient expression in *N. benthamiana* leaves infiltrated with a binary vector containing nnLuz. LBS- refers to two wild-type control plants; LBS + refers to three transgenics (Ly118.3, 5, 7) which were also the only three which were PCR + for multiple LBS genes. Error bars are standard deviations; the full screen of all 16 transgenics with statistics (P < 0.02) can be found in DataCommons (Spreadsheet Tab = Fig. S3) with the PCR gels aligned with all T0 plants in Supplemental File 1, Fig. S4.

Tomato explants were Agrobacterium transformed and regenerated with two different LBS constructs (Ly117 = pLSU4// HispS::H3H::CPH/ HygR, and Ly118 = pLSU2//NpgA:HispS:H3H:CPH/ KanR) were confirmed to be transgenic by a host of PCR screening as articulated in Supplemental File 1, Figure S4. Of the 16 regenerated Ly118 T0 lines, only three were PCR positive for the NpgA:HispS:H3H:CPH luciferin cycle genes. A functional screen was conducted by combining a simple leaf extract with luciferase that was confirmed to be transiently expressed in *N. benthamiana* (using authentic luciferin). A functional screen of the Ly118 T0 transgenics displayed a statistically significant elevated bioluminescence only for the three PCR positive lines (Ly118.3, 5 and 7; Fig. 4). However, the levels of bioluminescence were exceedingly low by comparison to other bioluminescence assay work with nanoluciferase for example. It was also noted that the level of luminescence in the luciferase-infiltrated *N. benthamiana* after luciferin addition rapidly decayed 25-fold to background bioluminescence in 13 min. Based on the level of luciferin supplied, this suggested either a rapid decline in luciferase activity, degradation of the luciferin substrate, or both.

**Fig. 5:**
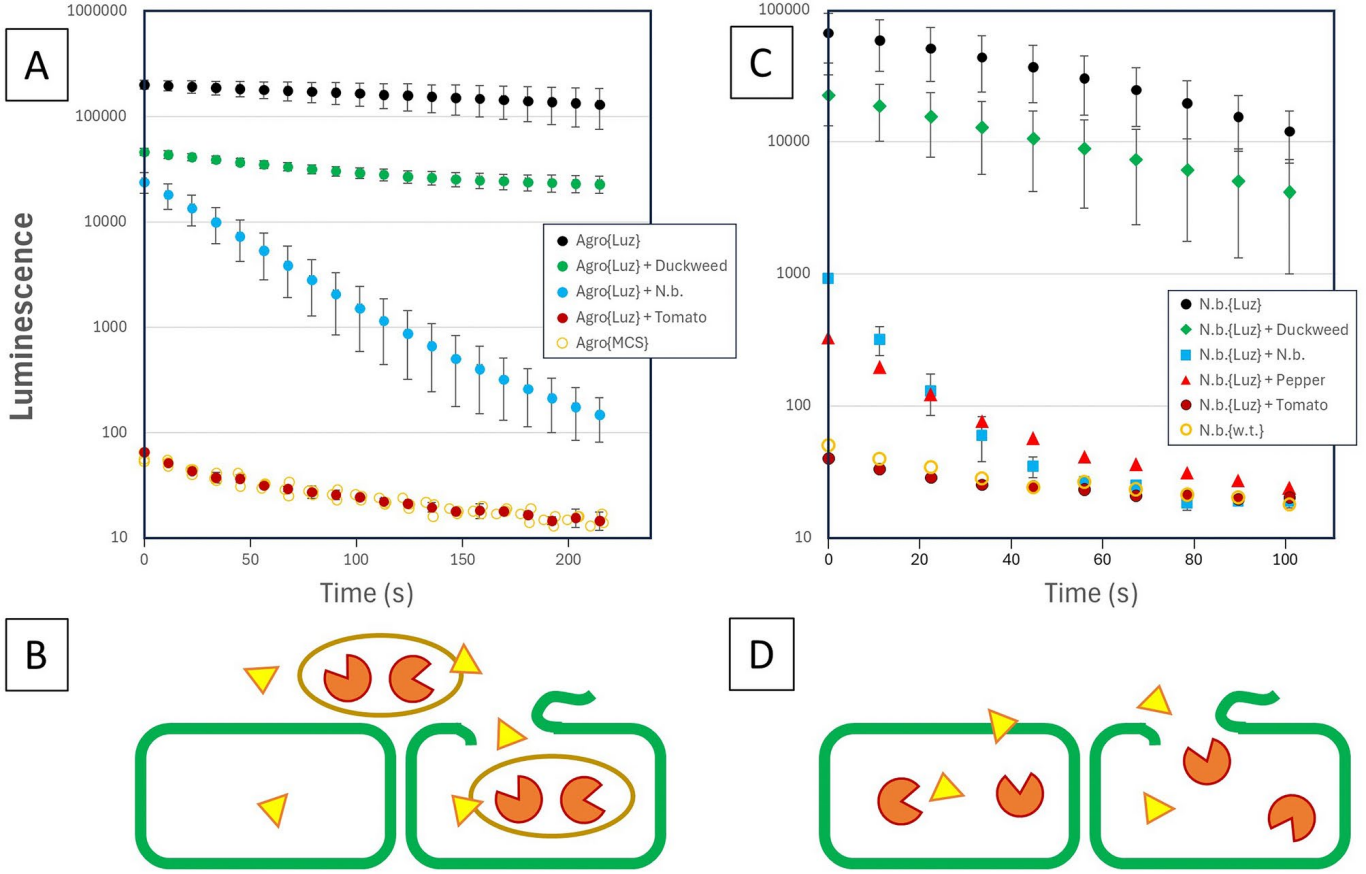
Quenching of nnLuz bioluminescence expressed in Agrobacterium and in *N. benthamiana* plant tissues. (**A**) Agrobacterium expressing nnLuz from binary vector in the presence of macerated tissues of duckweed, *N. benthamiana*, and tomato. (**B**) Schematic for the physical situation of panel **A** where the green represents intact and disrupted plant cells, luciferin is yellow triangles, and agrobacterium is the brown oval. (**C**) Bioluminescence after luciferin addition to macerated *N. benthamiana* tissue expressing nnLuz-intron in the presence of other plant tissues of duckweed, tobacco, pepper, tomato and wild-type *N. benthamiana*. (**D**) schematic for the physical situation of **B**. Error bars are standard deviations.

Based on our observation that plant promoters are active from Agrobacterium transformation binary vectors^22,23^, we screened various nnLuz-expressing constructs while including intron-containing nnLuz vectors as negative controls since Agrobacterium cannot process plant introns (Supplemental File 2, S2-2B). This led to the use of the simple Agrobacterium vectors containing the pLSU1//nnLuz-I{PIV216}, (Addgene #212187) and pLSU1//nnLuz without intron (Addgene #212183) as a biosensor reporters for expression of the luciferase in the plant and Agrobacterium respectively. Experiments with plant tissue were then conducted using a circular leaf punch within the 96-well plate, maceration with resuspended Agrobacterium and addition of luciferin (Fig. 5A,B).

As anticipated, the levels of bioluminescence in Agrobacterium were exceedingly high and comparatively constant over time as one would expect for a bioluminescence ‘glow assay’ for reasonably stable enzyme and substrate. In contrast, bioluminescence in the presence of macerated *N. benthamiana* dropped more than two orders of magnitude in under 4 min. Duckweed tissue displayed minimal decay, which may reflect in part less maceration. Tomato displayed a dramatic and apparently nearly instantaneous quenching of bioluminescence to background wild-type tomato tissue levels. Considering the luciferase is protected within the bacterial biosensor, the rapid decline largely reflects the instability of the luciferin substrate in the presence of the disrupted plant tissue.

To extend the observation of quenching to the mushroom luciferase expressed in plant tissue, transient expression of the intron-containing version of the luciferase, pLSU1//nnLuz-intron was Agrobacterium infiltrated into *N. benthamiana* to ensure plant-based expression. Leaf disks of this nnLuz infiltration layered on top of other plant tissue and assayed with 2-μL of 100 mM fresh luciferin using the pipette disruption method. These results confirmed the dramatic interference of the bioluminescence measurements caused by disrupted plant tissue (Fig. 5C,D). A layering of the nnLuz expressing *N. benthamiana* tissue over its own macerated wild-type tissue reduced bioluminescence by almost two orders of magnitude. The quenching by a tomato leaf disk was complete, with bioluminescence being indistinguishable from *N. benthamiana* leaf tissue control. Pepper leaf tissue was included as a comparative test for a related solanaceous plant species which was highly quenched but not fully quenched as observed for tomato—suggesting that disrupted tomato tissue releases metabolites that instantaneously quench the assay. A comparable mass of duckweed fronds (~ 3 mg) displayed minimal disruption, which could result either from absence of quenching species, or ineffective disruption using the pipette poking technique.

**Fig. 6:**
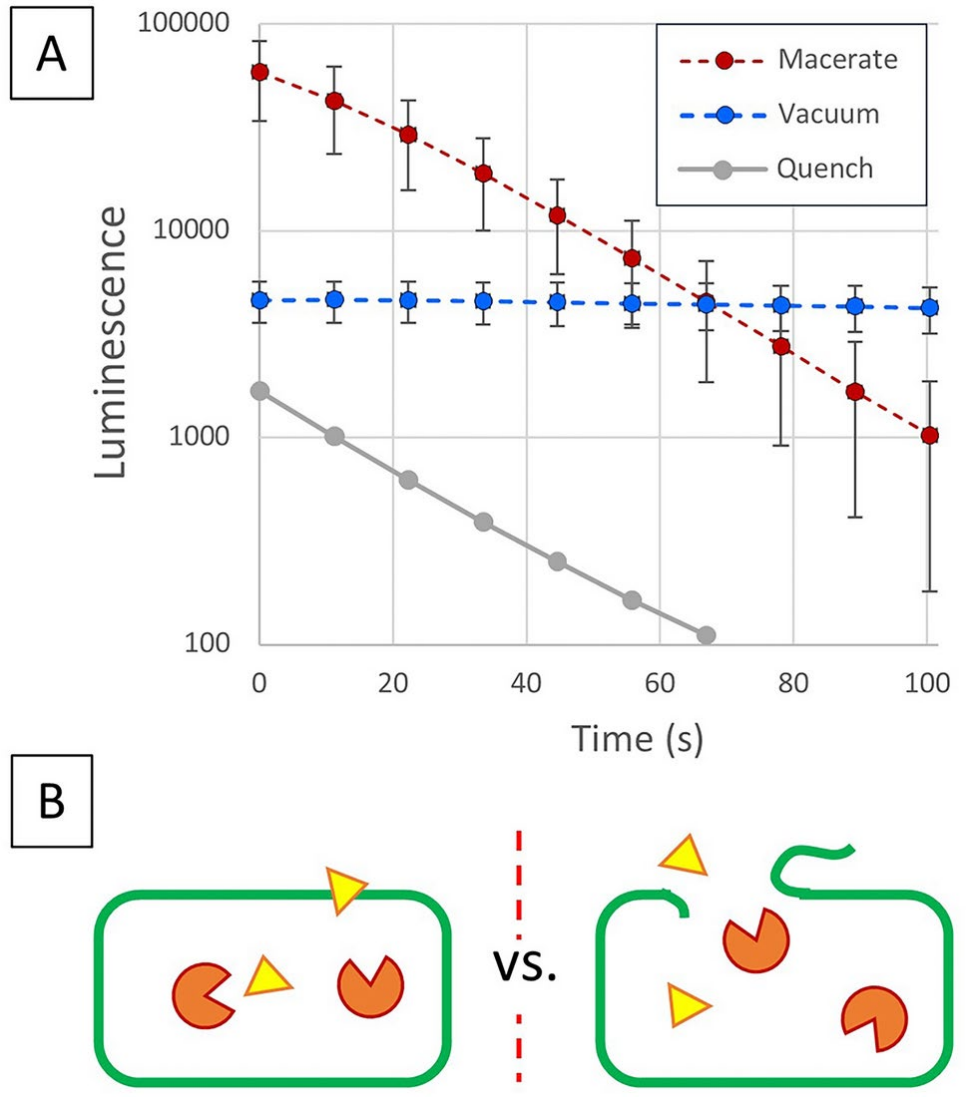
Bioluminescence comparison of luciferin introduced by vacuum infiltration with minimal plant tissue damage as compared to tissue maceration. (**A**) Expression of mushroom luciferase in *N. benthamiana* leaves at 3-dpi from syringe infiltration of Agrobacterium harboring nnLuz-intron with addition of the same amount of luciferin by either maceration of the tissue in the luminometer well or via vacuum infiltration in the 96-well plate. The same *N. benthamiana* tissue macerated with an additional leaf punch of wild-type *N. benthamiana* is included to illustrate elevated quenching. Error bars are standard deviations. (**B**) Schematic of the physical situation for panel **A** where vacuum infiltration must diffuse into the plant cells to reach the luciferase as compared to releasing the cellular contents and allowing luciferin substrate to enter disrupted cells.

To test for the mechanistic basis of quenching, an alternative strategy of exposing the plant tissue to with minimal tissue disruption was undertaken by vacuum infiltrating the luciferin into the leaf disk immediately prior to luminescence readings for comparison to the pipette maceration method. A dramatic difference was observed in the bioluminescence response of macerated versus vacuum infiltrated *N. benthamiana* expressing luciferase (Fig. 6) The exponential decay in bioluminescence for the disrupted tissue contrasts with a constant lower bioluminescence for the vacuum infiltrated luciferin. Keeping in mind the ~ 45 s delay in the reading start for the TECAN luminescence measurements, there is clearly an extremely high immediate consumption of luciferin that is not measured. Interestingly, the decay rate observed for the quenching with an additional underlayer of wild-type tissue parallels the exponential decay of the single macerated leaf punch. As depicted in the associated schematic (Fig. 6B), the treatment is intended to avoid plant cell disruption. As a result, the enzyme is presumed to be protected by the physiological environment, and the observed bioluminescence is a pseudo-glow assay as a result of being limited by the transport rate into the cells. The implication of this observation of mass transfer limited kinetics is further addressed in the discussion..

Of the six regenerated Ly117 transformed tomato plants that were successfully taken to T1 seed, five were lost due to mishandling during COVID constraints—rendering the seed non-viable. The remaining line Ly117.1 was self-crossed, and T2 seed were collected from nine offspring. The selectable marker for the Ly117 transformation was hygromycin; therefore, we could not use our kanamycin digital PCR probes for gene copy number. Sucker clones of these plants were maintained for over four years to provide tissue for screening. Nineteen regenerated Ly118 plants were produced, with 10 having the kanamycin resistance gene copy number of 1, and two with a gene insertion copy number of 2. Only three screened PCR positive for the LBS pathway genes (Ly118.3, 5 and 7). Only the Ly118.3 line was segregated to homozygous which had an original copy number of 2, so was segregated to two independent hemizygous lines (dPCR = 0.5), selfed and then identification of the single gene copy T3 insertion lines (dPCR = 1). The spreadsheet used for the tracking the segregation of these transgenic lines is available via Penn State’s DataCommons (https://www.datacommons.psu.edu/) for this publication.

**Fig. 7:**
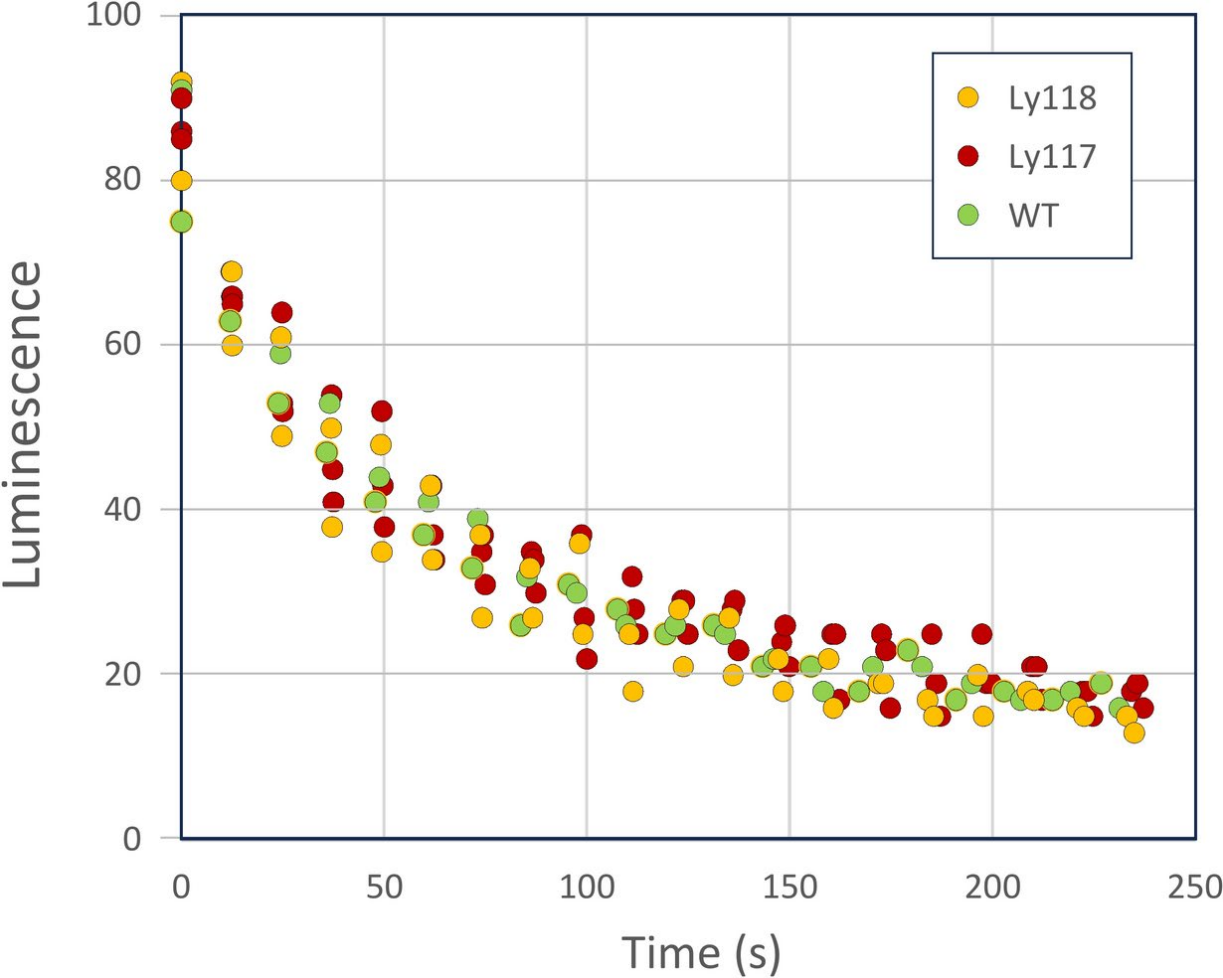
Screening for bioluminescence in homozygous, segregated tomato transformed with polycistronic luciferin biosynthesis pathway genes. Agrobacterium expressing luciferin was vacuum infiltrated as a biosensor for the presence of luciferin. The Agrobacterium harbors the binary vector containing the nnLuz-V4 luciferase that was improved by directed evolution^9^.

The homozygous lines of Ly118, and PCR positive segregants of Ly117 were subjected to the agrobacterium biosensor maceration method with the results being indistinguishable from background (Supplemental file 1, Fig. S6). Attempts to transiently express the luciferase using agrobacterium infiltration were also not successful, although we note tomato is orders of magnitude less efficient at transient expression than *N. benthamiana*. If expression could be improved, the addition of caffeic acid could help distinguish a rate limitation between either HispS or H3H. Based on the bioassay refinement minimizing tissue disruption using vacuum infiltration, and use of agrobacterium as a biosensor, we undertook a final effort to quantify possible luciferin in these LBS transgenics. To do this, we created a binary vector based on the recently reported enhanced stability luciferase, pLSU1//35s:nnLuz-V4^9^. A screen of the transgenic Ly117 and Ly118 lines using this Agrobacterium biosensor approach failed to give bioluminescence levels different from background wild-type control (Fig. 7).

## Discussion

The long-term goal of our research was to create a transgenic tomato plant that produced luciferin that would allow monitoring of virus movement for a genetically engineered begomovirus expressing the complementary luciferase. Such an experimental system would provide quantitative data to refine our efforts to model virus proliferation and movement in plants (https://etda.libraries.psu.edu/catalog/19293nst31). This project was supported by DARPA as part of a program to develop insect-delivered deconstructed viruses for crop protection (https://www.darpa.mil/program/insect-allies). Such a technology would allow for rapid deployment of ‘gene therapy’ in the field in response to crop needs rather than current GMO strategies. Towards this end, pseudo-polycistronic transformation constructs were created for the luciferin biosynthesis and recycle pathway that excluded the luciferase. These constructs were demonstrated to produce luciferin *in planta* based on transient expression from Agrobacterium delivery of these genes. Transgenic tomato plants were generated with these constructs with initial indications of luciferin biosynthesis, albeit very low levels of bioluminescence. Subsequent generations of segregated transgenic plant selection to a homozygous state resulted in a loss of measurable luciferin biosynthesis and a greater appreciation for the critical role of luciferin substrate stability in creating an LBS + transgenic plant phenotype.

The creation of separate components for luciferin and enzyme expression led to further studies to understand the kinetics of this reaction. Notably, the luciferin substrate was particularly unstable in the presence of plant cell extracts and even buffer resuspended agrobacterium—complicating efforts to assay biosynthesis. This work also revealed that tomato tissue extracts are particularly active at quenching the bioluminescent assay—making it nearly impossible to screen for transgenic produced luciferin. Keeping in mind that the light reaction is on par with the energy of ATP hydrolysis, the observed decay rates of the bioluminescent reaction over a few minutes is not unexpected, where even synthetic assay luciferins require careful handling^24^. The instability of natural luciferins is widely known, where different strategies have evolved to overcome this limitation to storing sufficient energy to produce visible amounts of light. In dinoflagellates, there are a diversity of luciferin binding proteins that provide protection from luciferin oxidation^25^. In insects, the luciferin is bound within a protein complex referred to as the SBF (substrate binding fraction) as an evolution to achieve the same goal^26^. Consistent with a need to protect the energy stored in luciferin, for marine environments where it is estimated that more than half of the organisms utilize bioluminescence, it is common for the luciferin and luciferase to form a stable complex referred to as the photoprotein^27^. This complex allows rapid activation for a flash reaction rather than a typical substrate-enzyme kinetics. Such a storage / protective mechanism for the mushroom luciferin (3-hydroxyhispidin) has not yet been identified. Most bioluminescent mushrooms luminesce, even in the light, suggesting a metabolic role for dissipation of oxidative species; however, a recent report for circadian-mediated bioluminescence revealed all components undergoing circadian control^28^. In either case, the biomolecular mechanism may be rate-limited by the formation of the luciferin in a point-of-use reaction, such that there is no requirement for luciferin storage. In the absence of luciferase, mushroom luciferin may simply be dissipated by the diversity of antioxidative systems such as superoxide dismutase, catalase, and various peroxidases^29^.

These results provide insight into future path forward to create a convenient transgenic for in vivo reporting with this rapidly developing pathway. Since initial study on the mushroom luciferase pathway has included the luciferase as the basis of creating ‘glowing plants’^5,6^, it might be logical to screen for high luciferin production in these plants, and then knock out the luciferase. However, it is not unlikely that such a strategy could result in toxicity or silencing of the LBS pathway. It might instead be logical for the plant to express the luciferase, and complement with luciferin biosynthesis. In the absence of finding a luciferin binding protein for 3-hydroxyhispidin, an alternative would be to over-express genes for hispidin accumulation. Notably, hispidin is an antioxidant that can accumulate to high levels in non-bioluminescent mushrooms^30^, and a greatly improved transgenic pathway has recently been described for hispidin production that eliminates the need for the massive (5.1-kb) HispS as well as its associated NpgA^9^. A transgenic plant designed for elevated hispidin and oxyluciferin recycle with complementation by H3H for luciferin production could provide for substrate accumulation without generating the reactive luciferin and negative selection pressure.

The work presented here provides some discussion about the use of the mushroom luciferase as a quantitative assay. Ideally, a bioluminescent assay is conducted in the ‘glow regime’, where the luciferin substrate is much higher concentration than the luciferase K_m_. Based on saturation kinetics, bioluminescence would be proportional to the enzyme turnover number (ε∙ v_max_).

$${B}_{ez}=\varepsilon \left(\frac{{v}_{max} \bullet S}{{K}_{m}+S}\right) \cong \varepsilon \bullet {v}_{max}$$

An advantage of a kinetically limited glow assay is that the bioluminescence level is proportional to the amount of enzyme. In addition, the concentration of substrate can vary considerably—including non-photon producing degradation without affecting the assay. A disadvantage of the glow regime is the requirement for relatively high luciferin concentration of this expensive substrate. The ongoing improvements into the overall pathway, including luciferase, are a major improvement, although they do not address the instability of the substrate. While zero order kinetics does result in a linear decline in substrate, dS/dt = constant, that decline is not reflected in a linear decline in luminescence. The observation of a comparatively constant luminescence for intact Agrobacterium (Fig. 5) demonstrates the assay substrate concentration used is sufficient to sustain a prolonged luminescence, therefore, the observed decline in bioluminescence is not depletion by the luciferase reaction. Numerous lines of evidence support the observation that the decrease in bioluminescence is substrate degradation. A decline in enzyme levels can also contribute to the observed drop in bioluminescence, however, a simple test of refreshing substrate resulted in full recovery of luminescence (Supplemental File 1, Figure S5A) showing that the enzyme remained protected within the agrobacterium. Further substantiation for a role of cellular components facilitating the substrate degradation are observed for Agrobacterium resuspension in cell disruption buffers which dropped luminescence quickly as compared to resuspension in the culture centrifugation supernatant (Supplemental File 1, Figure S5B). This indicates that leakiness from the cell (as well as tissue extracts) contributes to the rapid breakdown of mushroom luciferin. Noting that the reported estimated K_m_ for the mushroom luciferase is 25 mM [^9^; reference supplementary File 1, Fig. S6)] and our assay concentration of about 9 mM, the luminescent reaction is in the linear kinetic region (resulting in a first order exponential decay), the half-life for the 3-hydroxyhispidin under these assay conditions is about 17 s—illustrating the challenge of utilizing this luciferase for a kinetic assay (calculations provided in the DataCommons archive spreadsheet). Our handling of the luciferin substrate in small aliquots, − 80 °C in DMSO reflects the rigorous handling necessary to achieve reproducible results.

Vacuum infiltration of substrate with minimal cell disruption provides additional insight into the bioluminescence behavior. When entry into the cells is slower than the rate of consumption, the bioluminescence is proportional to the rate of luciferin transport into the cells.

$${B}_{ez}={(k}_{S}A)\left(S-{S}_{in}\right) \cong {(k}_{S}A)\left(S\right)$$

The observation of very constant luminescence for vacuum infiltration into the leaf (Fig. 6) is consistent with such a mass transfer limited bioluminescence. Under these conditions, the internal substrate concentration within the cell (S_in_) is reacted to be essentially zero resulting in a bioluminescence that is proportional to the permeation of substrate (k_S_A, apparent mass transfer coefficient and area). This pseudo-glow assay is not reflective of saturation of the luciferase kinetics, but instead due to a transport rate that is sufficiently slow to result in a comparatively constant apoplastic concentration of luciferin, and a constant driving force for entry into the cell. Therefore, while the luminescence assay provides for more stable quantification of the presence of luciferin, it does not provide a quantitative basis for the levels of luciferase gene expression. Because bioassays can involve mass transfer limited kinetics simultaneously with kinetic limitation, the observed effective enzyme kinetics can be altered relative to intrinsic kinetics.

The choice of tomato for the transgenics was dictated by the funding of crop-relevant research associated with a goal of assessing viral movement based on bioluminescence. In contrast to the success of using either individual or polycistronic transient expression in *N. benthamiana* we were not able to obtain quantitative bioluminescence assay measurements above background for tomato. While we initially attributed this difficulty to our experience with transient expression levels in tomato that are several orders of magnitude lower than *N. benthamiana*, the issues of assay quenching also likely contributed to these difficulties. As this technology is translated to non-model systems, the challenge of adapting from model systems to crops of commercial interest needs to be kept in mind.

## Methods

### Cloning

#### Monocistronic LBS constructs

Individual luciferin biosynthesis genes (with the exception of NpgA) were kindly provided by Karen Sarkisyan in advance of publication^6^ and cloned into the highly compact pLSU-2 *Agrobacterium* binary vector^31^ using Gibson Assembly. NpgA codon-optimized for expression in tomato was synthesized by TWIST Bioscience.The pLSU-2 backbone sequence is available on Genbank ID = OR695069.1. The individual genes were readily assembled by first PCR amplifying a 0.4 kb 35S promoter and TMV Ω’ 5’ UTR from pX028^6^, the coding sequence for the gene (pX028, pX019, and pX020 for HispS, H3H, and CPH, respectively), and the NOS terminator from pBI121^32 ^with homology extensions. The pLSU-2 backbone was linearized using a KpnI digest and rSAP-mediated dephosphorylation of 5’ ends. Each set of fragments for individual gene assembly, including the linearized pLSU-2 backbone, were incubated with NEB Hi-Fi Assembly Master Mix (NEB, cat.# E2621L) and used to transform TOP10 *E. coli*. The specific primer sequences for each of the cloned genes are provided in Supplemental File 2, S2-1C.

#### Polycistronic LBS constructs

Since these vectors are designed for stable transformations, they contain selectable markers: pLSU2 contains kanamycin resistance, and pLSU4 contains hygromycin resistance. To avoid homologous repeats that might cause genetic instability, a set of six non-homologous versions of the intein-F2A peptides that share no more than 11 continuous nucleotides of homology with the original published sequence were designed using the non-repetitive parts calculator^33^. Of the set of seven, three, including the original, were synthesized for this study. These sequences are provided in Supplementary File 2, S2-2C. The enzyme-modifying role of the heterologous phosphopantetheinyl-transferase had conflicting reports on its requirement (Karin Sarkisyan, personal communication), therefore, creating vectors without NpgA was a priority. All vectors used a 0.4-kb CaMV 35S promoter and TMV Ω’ 5’ UTR. The specific primer sequences for each of the cloned genes are provided in Supplemental File 2, Table S2-1C. The correct insertion into the destination vector for all polycistronic constructs was confirmed using ‘bracket PCRs’ with {pLSU2-4 5’ Fwd} and {LBS17} for the most upstream junction and {LBS18} and {pLSU-1 3’ R} for the most downstream junction as well as Sanger sequencing.

**Ly115 = pLSU4/P35S(0.4 kb)[TMV Ω’]:H3H:oIntF2A:CPH:Tnos** was created using Gibson Assembly. Starting materials were the pLSU-2/P35S(0.4 kb)[TMV Ω’]:H3H:Tnos, pLSU-2/P35S(0.4 kb)[TMV Ω’]:CPH;Tnos, pJET1.2/oIntF2A, and pLSU-4. A 35S promoter + H3H fragment, oIntF2A fragment, and CPH + NOS terminator fragment were amplified from their respective source plasmids with homology extensions. The P35S(0.4 kb)[TMV Ω’]:H3H fragment was amplified using primers {LBS13} and {LBS36}, the oIntF2A fragment with {LBS35} and {LBS38}, and the CPH:Tnos fragment with {LBS37} and {LBS14}. The pLSU-4 backbone was linearized with a KpnI digest and rSAP-mediated dephosphorylation of 5’ ends. The fragments for assembly, including the linearized pLSU-4 backbone, were incubated with NEB Hi-Fi Assembly Master Mix (NEB, cat.# E2621L) and used to transform TOP10 *E. coli*.; GenBank accession number PQ667060.

**Ly117 = pLSU4/P35S(0.4 kb)[TMV Ω’]:HispS:nrIntF2A-1:H3H:oIntF2A:CPH:Tnos** was created using Gibson Assembly. Starting materials were pLSU-2/P35S(0.4 kb)[TMV Ω’]:HispS:Tnos, pLSU-2/P35S(0.4 kb)[TMV Ω’]:H3H:oIntF2A:CPH;Tnos, pJET1.2/nrIntF2A-1, and pLSU-4. A 35S promoter + HispS fragment, nrIntF2A-1 fragment, and H3H:oIntF2A:CPH:Tnos fragment were amplified from their respective source plasmids with homology extensions. The P35S(0.4 kb)[TMV Ω’]:HispS fragment was amplified using primers {LBS13} and {LBS43}, the nrIntF2A-1 fragment with {LBS44} and {LBS45}, and tbe H3H:oIntF2A:CPH:Tnos fragment with {LBS46} and {LBS14}. The pLSU-4 backbone was linearized with a KpnI digest and rSAP-mediated dephosphorylation of 5’ ends. The fragments for assembly, including the linearized pLSU-4 backbone, were incubated with NEB Hi-Fi Assembly Master Mix (NEB, cat.# E2621L) and used to transform TOP10 *E. coli*. A detailed cloning strategy with primer analysis and construct schematic is provided in Supplemental File 2, S2-1A; GenBank accession number PQ667061.

**Ly118 = pLSU2/P35S(0.4 kb)[TMV Ω’]:NpgA:nrIntF2A-2:HispS:nrIntF2A-1:H3H:oIntF2A:CPH:Tnos:** The full LBS pathway including NpgA was similarly constructed as Ly117 using Gibson Assembly. Starting materials were pLSU4/P35S(0.4 kb)[TMV Ω’]:HispS:nrIntF2A-1:H3H:oIntF2A:CPH:Tnos, pLSU-4/P35S(0.4 kb)[TMV Ω’]:NpgA:Tnos, pJET1.2/nrIntF2A-2 and pLSU-2. A 35S promoter + NpgA fragment, nrIntF2A-2 fragment, and HispS:nrIntF2A-1:H3H:oIntF2A:CPH:Tnos fragment were amplified from their respective source plasmids with homology extensions. The P35S(0.4 kb)[TMV Ω’]:NpgA fragment was amplified using primers {LBS13} and {LBS54}, the nrIntF2A-1 fragment with {LBS53} and {LBS56}, and tbe HispS:nrIntF2A-1:H3H:oIntF2A:CPH:Tnos fragment with {LBS55} and {LBS14}. The pLSU-4 backbone was linearized with a KpnI digest and rSAP-mediated dephosphorylation of 5’ ends. The fragments for assembly, including the linearized pLSU-4 backbone, were incubated with NEB Hi-Fi Assembly Master Mix (NEB, cat.# E2621L) and used to transform TOP10 *E. coli*.; GenBank accession number PQ667062.

***pLSU1/t35s[]:nnLuz:NosT***. To create pLSU1/t35s[]:nnLuz:NosT, The mushroom luciferase of *N. nambi* (nnLuz) was amplified by PCR from pX018 with restriction overlap primers {nnLuzIF} and {nnLuzIR}, digested using KpnI and BamHI, and ligated into pLSU1/t35s[]:MCS:NosT, cut with the same enzymes resulting in pLSU1/t35s[]:nnLuz:NosT. Addgene ID = 212183.

***pLSU1/t35s[]: nnLuz-Intron{PIV-216}:NosT.*** The intron-containing version of the mushroom luciferase nnLuz-I was constructed using the Gibson assembly with pJET1.2/blunt as the destination vector and TOP10 *E. coli* as the cloning strains. The mushroom luciferase was kindly provided by Karen Sarkisyan in pX019 in advance of publication^6^, which is codon-optimized for expression in *Nicotiana benthamiana*. The intron chosen was the potato PIV2 Intron (intron 2 of the *Solanum tuberosum* ST-LS1 gene; Genbank ID X04753.1) which contains stop codons in all three reading frames to assure premature translation termination if expressed in *Agrobacterium*. The PIV2 intron has been used successfully for *Agrobacterium* transient expression of both the GUS and GFP reporter genes in both monocots and dicots^17,22,34^. The placement of the intron was based at nucleotide 216 on considerable bioinformatic analysis because no experimentally determined protein structure for nnLuz was available. Analysis to determine an optimal intron location included a combination of amino acid sequence comparison of homologs in other closely related fungal species and protein structure prediction to find an N-terminal conserved region that could serve a critical function of the enzyme. Homologs to nnLuz were found by querying its amino acid sequence against the NCBI BLAST translated nucleotide database using the tBLASTn method. Only homologs in known bioluminescent fungal species were selected for analysis. Conserved regions among these homologs were found via protein multiple sequence alignment in Benchling (benchling.com/) using Clustal Omega (ebi.ac.uk/Tools/msa/clustalo/) methodology (Figure S2-2B.1). Secondary structure prediction of Luz (Figure S2-2B.2) was generated using the PredictProtein web tool^35^. Intron insertions in Luz were checked in-silico for the absence of off-target splice sites using the NetGene2 web server^36^ with settings for *Arabidopsis thaliana* (Figure S2-2B.3), and the absence of inhibitory RNA stem-loops close to the intron splice sites using the ViennaRNA RNAfold web server (rna.tbi.univie.ac.at/). The 5’ and 3’ fragments of nnLuz CDS were amplified from pX018 (provided by Planta LLC ahead of publication Mitiouchkina et al.^5^; using overlap primer pairs {nnL5}/{nnL6} and {nnL7}/{nnL8}, respectively. The PIV2 intron was amplified from the pEAQ-GUS plasmid^37^ with overlap primers {nnL9} and {nnL10} as listed in Supplemental File 2,  Table S2-1C. For ligation into the pLSU1 drop in expression vector, nnLuz-I{PIV-216} was amplified by PCR from pJET1.2/nnLuz-I{PIV-216} with a 5’ XhoI site extension and a 3’ EcoRI site extension using primers {nnL11} and {nnL12} and then ligated with the pLSU1/t35s:MCS:nosT backbone and digested with XhoI and EcoRI, to create pLSU1/t35s[]:nnLuz-I{PIV-216}:NosT. Addgene ID = 212187.

### Transient expression/agrobacterium infiltration

The agrobacterium transient expression was based on syringe infiltration procedure^13^. Agrobacterium containing the binary vector is grown from cryostock on selective LB solid media, and then grown overnight to an OD_600_ < 1.0 on liquid media. The liquid culture is pelleted, and resuspended to an OD_600_ = 0.6 in a 10 mM MES, 10 mM MgCl_2_ infiltration buffer (Supplemental File 2, S2-3A). Incubate for 1-h at 25 °C in 0.1 mM acetosyringone to activate T-DNA transfer. Surfactant 0.02% Silwet L-77 added to facilitate gentle syringe infiltration through the stomata on the underside of 4-week old *N. benthamiana* plants that have been growth with 12-h photoperiod and hardened for at least two days at room humidity outside of the incubator to reduce necrosis. Measurements of gene expression are ideally conducted at 3-dpi (days post infiltration). Mixed culture infiltrations are generally conducted at equal ratios.

### Tomato plant transformation, regeneration, selection

The tomato cotyledon transformation protocol was adopted (with slight modifications) from Arshad et al., 2014^38^. Tomato seeds of *Lycopersicon esculentum* (var. Florida Lanai) were surface sterilized with 5% v/v commercial bleach (6% w/v Na-hypochlorite) with 1-drop of Tween-20 surfactant per 100 mL and germinated on solidified hormone-free 1/2 strength MS salts media^39^. Agrobacterium is grown on bacterial selective media (50 mg/L kan, 20 mg/L rifampicin) for ~ 2 days, and resuspended in 1/2 strength MS salts liquid with 20 µM acetosyringone for vir gene activation. Cotyledons and hypocotyls were excised at ~ 9 days, incubated with activated *Agrobacterium* suspension for 20 min with gentle shaking, blotted dry, and cultivated in the dark for 48 h on sterile 1/2 strength MS-soaked filter paper. Explants are then washed with 1/2 MS salts liquid medium containing 500 mg/L cefotaxime and plated on full strength MS salts agar plant selection media (MS salts agar with 500 mg/L cefotaxime, 150 mg/L ticarcillin/clavulanate, 2 mg/L zeatin, and 0.1 mg/L indole-3-acetic acid): 100 mg kanamycin / L selection for the Ly118 transformation vector, and 20 mg hygromycin / L selection media for the Ly117 construct. Explants are transferred bi-weekly to fresh selection media to prevent agrobacterium overgrowth and observe plantlet regeneration. Independent transformants (based on individual explants) based on shoot primordia are transferred to shoot induction media with reduced zeatin: 0.1 mg/L zeatin, and 0.1 mg/L indole-3-acetic acid (with continued antibiotics for Agrobacterium; 500 mg/L cefotaxime, 150 mg/L ticarcillin/clavulanate, and transgene selection 100 mg/L kanamycin). Once regenerated plants have discernable shoot, they are moved to root induction media which further reduces plant hormones to 0.05 mg/L indole-3-butyric acid and continued 500 mg/L cefotaxime, and transgene selection (100 mg/L kanamycin or 20 mg/L hygromycin as appropriate). Rooted transgenics are then transferred to soil and grown for several weeks (while being tested for transgene for PCR, including a primer set for the Agrobacterium binary vector *E.coli* KanR selectable marker nptI promoter (Fwd = CCACGTTGTGTCTCAAAATCTC, Rev = AACACCCCTTGTATTACTGTTTATG) as a control to assure absence of the Agrobacterium transformation vector.

### Transgenic tomato plant characterization

PCR screening for transgene insertion (selectable markers NptII / HPH and LBS genes) was undertaken using the primers as provided in Supplemental File 2, Table S2-1C. Gene copy number insertion was assessed only for Ly118 based on the probes developed for NptII selectable marker (dPCR,^40^). dPCR was performed by the Penn State Genomics Core Facility at University Park using the QuantStudio 3D Digital PCR System according to the manufacturer’s protocol (ThermoFisher) where tomato PROSYSTEMIN (SISYS) is used for the reference gene based on primers (Fwd = GCAATATCAAGAGCCCCGTC, Rev = ATGTGTGCTAAGCGCTCC) to produce a 91 bp amplicon. The probes for kanamycin selectable marker (nptII, Fwd = TTGCCGAATATCATGGTGGA, Rev = TCAGCAATATCACGGGTAGC) produce a 113-bp amplicon. The QuantStudio 3D Digital PCR System was used in this work operated at 60 °C using HEX™-labeled probe for nptII (5’HEX/CCGGCCACA/ZEN/GTCGATGAATCC/3’IABkFQ double-quenched with ZEN and Iowa Black Hole Quencher) and FAM™-labeled probes for SISYS (5′6-FAM/TGCAACATC/ZEN/CTTCTTTCTTCTCGTG/3’IABkFQ). Tissue samples were predominantly leaf, though some fruit tissue was also used. Tissue was frozen in liquid nitrogen, then ground in a BioSpec mini-beadbeater. DNA is then extracted using the MasterPure™ Complete DNA and RNA Purification kit according to manufacturer instructions to provide a minimum required yield of 300 ng/μL (average of > 1000 ng/μL was obtained). DNA concentrations were determined by Qubit dsDNA assay and then diluted to 14 ng/mL (based on genome size). Probe (8 mL) and primer (18 mL) at 4 mM were combined with the reaction mix: 7.25 mL dPCR master mix, 2.9 mL primer/probe mix, and 3.85 mL DNA sample. While the protocol for the dPCR provides a recommended dilution range based on the genome size, we found that far less serial dilution could be implemented to minimize chip use while still providing for accurate copy number determination.

### Luciferase assay

Mushroom luciferin (3-hydroxyhispidin) was kindly provided by Drs. Karen Sarkisyan and Ilia Yampolsky (Planta LLC, Moscow). The luciferase assay evolved considerably during the execution of this research. The initial procedure was adapted from the nanoluciferase assay. This involved taking a 7-mm diameter (#7) *N. benthamiana* or tomato leaf punch. Tissue samples were placed immediately on ice in a 2 mL cryotube with 2.4 mm diameter steel grinding beads and transferred into liquid nitrogen. Sample is intermittently ground in a Mini BeadBeater 24 Cell Disruptor by BioSpec for 5 s with transfer back to liquid N_2_ until the sample is visually ground followed by addition of CCLR extraction buffer at 2-mL per g fresh weight. The sample was then centrifuged in mini-table top centrifuge (Denville, mini-mouse), and placed on ice. 10 µL of leaf extract is combined with the same volume 0.1-mM mushroom luciferin substrate for measurement of bioluminescence in a luminometer (Promega, Glomax 96). Subsequent assays involved Eppendorf tube grinding of a 200-mg tissue sample, table-top spin and 20-μL supernatant plus 10-μL of luciferin substrate (maintained on ice in DMSO) with immediate single sample reads on a TECAN (Infinite M Plex Pro 200).

The refined procedure involves working with aliquots of 0.1-mM luciferin in DMSO distributed in 1 to 5-L and stored in epitubes at − 80 °C. These are re-suspended at 10 × dilution with phosphate buffer (1 M, pH = 7.5) immediately before use and kept on ice. Note that we had recently developed a kinetic assay extrapolating to the − 45 s initial condition as the basis of the utilization of the nnLuz-intron in conjunction with a screen of different promoters (Available at DataCommons 10.26208/GQ35-TQ59). Several specific assay techniques of note are as follows:

***N. benthamiana leaf disc***: A 2-mm (#2) leaf punch is taken through the underside of the leaf and placed into the well of a 96-well luminescence plate using a stick applicator. A 20-μL bead of CCLR buffer (Promega, Cat# E1531; Supplemental File 2, S2-3B) is placed on the leaf disk and 2-μL of 10 mM luciferin substrate followed by maceration by poking the tissue ~ 20 times and immediate TECAN luminescence time course.

**Agrobacterium luciferin biosensor**: *Agrobacterium tumefaciens* Cys32 auxotroph^41^containing binary vectors with the luciferase driven by plant promoters that are active in Agrobacterium express the active luciferase. Pelleted cultures resuspended in LB media at OD_600_ between 2 and 3 will luminesce in the presence of luciferin. Testing of agrobacterium alone involved addition of 2-μL (10-mM luciferin). Plant tissue quenching studies involved adding 20-μL of the agrobacterium suspension on top of the leaf disk, addition of 2-μL luciferin and macerating with the pipette tip. Plant tissues included tomato (*Lycopersicon esculentum*, var. Florida Lanai)^42^, Duckweed (*Lemna minor*), *Nicotiana benthamiana* acyl-sugar knockout^43^, and Jalapeno pepper (*Capsicum annuum*). Vacuum infiltration involved placing the 96-well plate into a 3-gal vacuum chamber (Ablaze) and pulling a 25 inHg vacuum for 30 s prior to reading in the TECAN luminometer.

**Luciferin vacuum infiltration** is executed in the same procedure as the Agrobacterium biosensor only CCLR and luciferin are added prior to the vacuum procedure.

## Supplementary Information

Supplementary Information 1.

Supplementary Information 2.

## Data Availability

Data corresponding to figures and associated statistics has been deposited in the Penn State Data Commons (www.datacommons.psu.edu) at URL 10.26208/WF41-JW81).
