# Supplementary material for: Expression of a multigene mushroom luciferin biosynthesis pathway as a pseudo-polycistron in plants
Source: Sci Rep. 2025 Jul 14;15:25385. doi: 10.1038/s41598-025-98717-2 (PMC12259908; doi:10.1038/s41598-025-98717-2)
Supplement: Supplementary file 1 — Supplementary Information 1. [file 41598_2025_98717_MOESM1_ESM.pptx]

## Slide 1
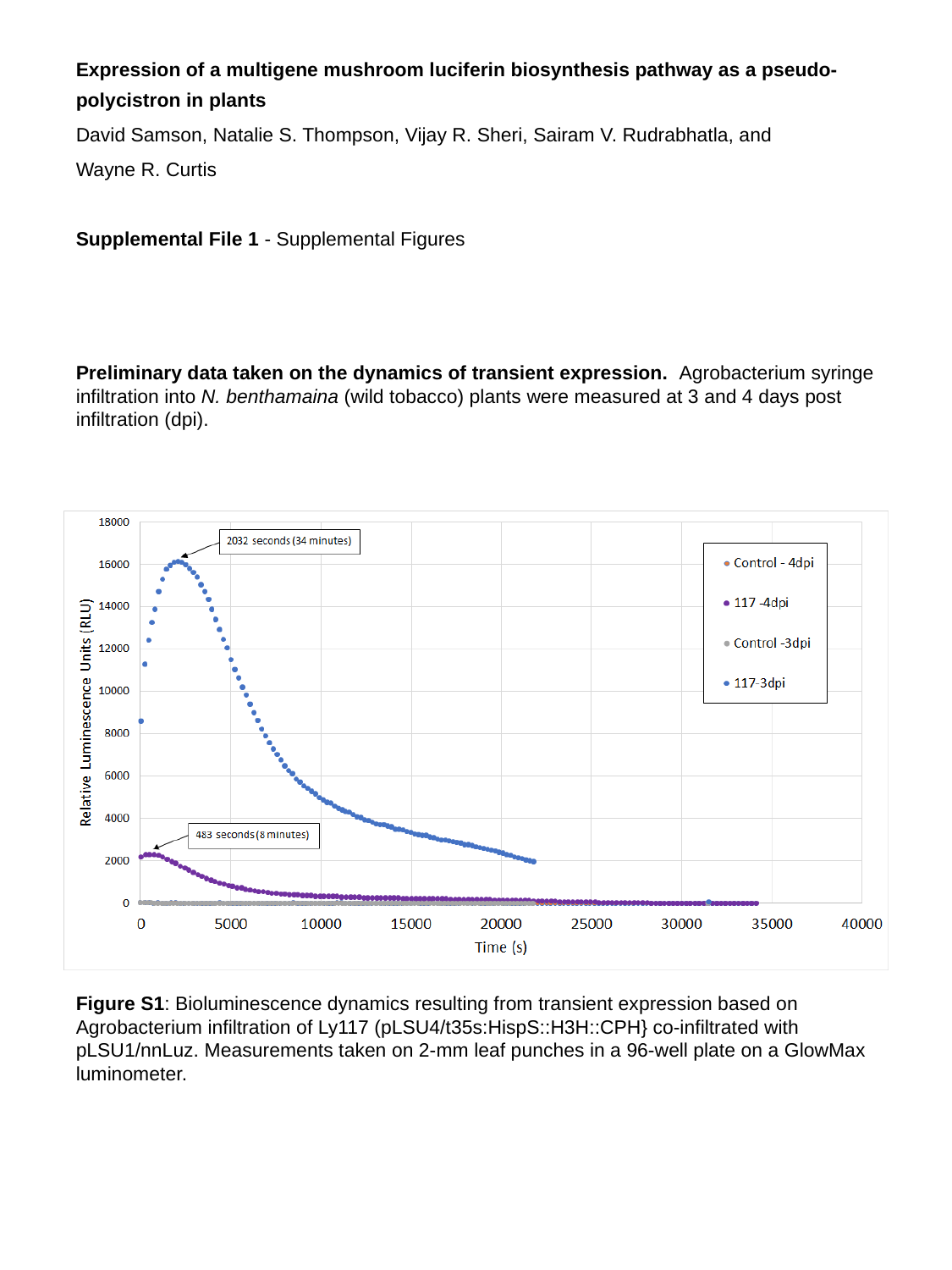

Expression of a multigene mushroom luciferin biosynthesis pathway as a pseudo-polycistron in plants
David Samson, Natalie S. Thompson, Vijay R. Sheri, Sairam V. Rudrabhatla, and
Wayne R. Curtis
Supplemental File 1 - Supplemental Figures
Preliminary data taken on the dynamics of transient expression. Agrobacterium syringe infiltration into N. benthamaina (wild tobacco) plants were measured at 3 and 4 days post infiltration (dpi).
Figure S1: Bioluminescence dynamics resulting from transient expression based on Agrobacterium infiltration of Ly117 (pLSU4/t35s:HispS::H3H::CPH} co-infiltrated with pLSU1/nnLuz. Measurements taken on 2-mm leaf punches in a 96-well plate on a GlowMax luminometer.

## Slide 2
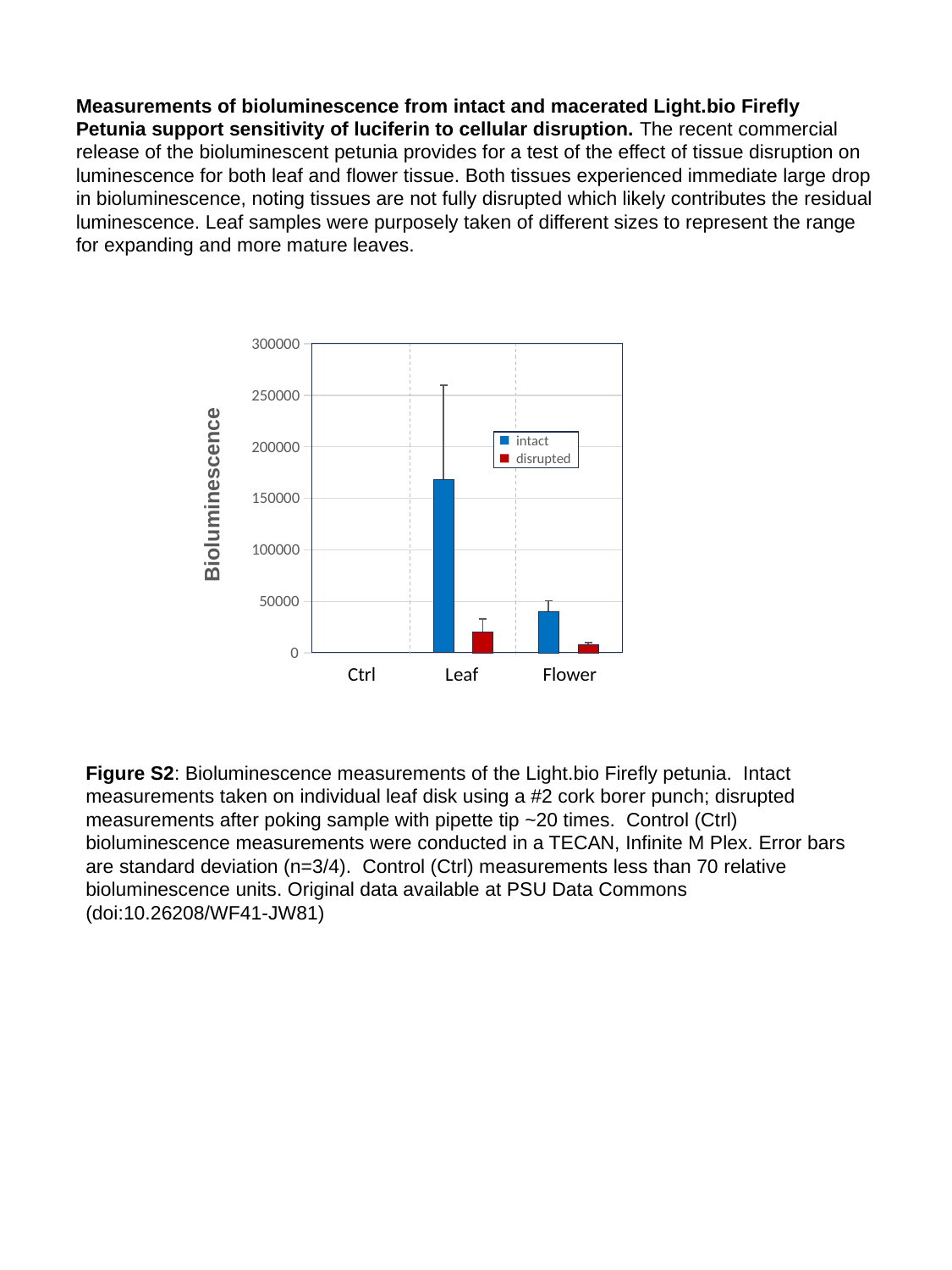

Measurements of bioluminescence from intact and macerated Light.bio Firefly Petunia support sensitivity of luciferin to cellular disruption. The recent commercial release of the bioluminescent petunia provides for a test of the effect of tissue disruption on luminescence for both leaf and flower tissue. Both tissues experienced immediate large drop in bioluminescence, noting tissues are not fully disrupted which likely contributes the residual luminescence. Leaf samples were purposely taken of different sizes to represent the range for expanding and more mature leaves.
300000
250000
intact
disrupted
200000
Bioluminescence
150000
100000
50000
0
Ctrl
Leaf
Flower
Figure S2: Bioluminescence measurements of the Light.bio Firefly petunia. Intact measurements taken on individual leaf disk using a #2 cork borer punch; disrupted measurements after poking sample with pipette tip ~20 times. Control (Ctrl) bioluminescence measurements were conducted in a TECAN, Infinite M Plex. Error bars are standard deviation (n=3/4). Control (Ctrl) measurements less than 70 relative bioluminescence units. Original data available at PSU Data Commons (doi:10.26208/WF41-JW81)

## Slide 3
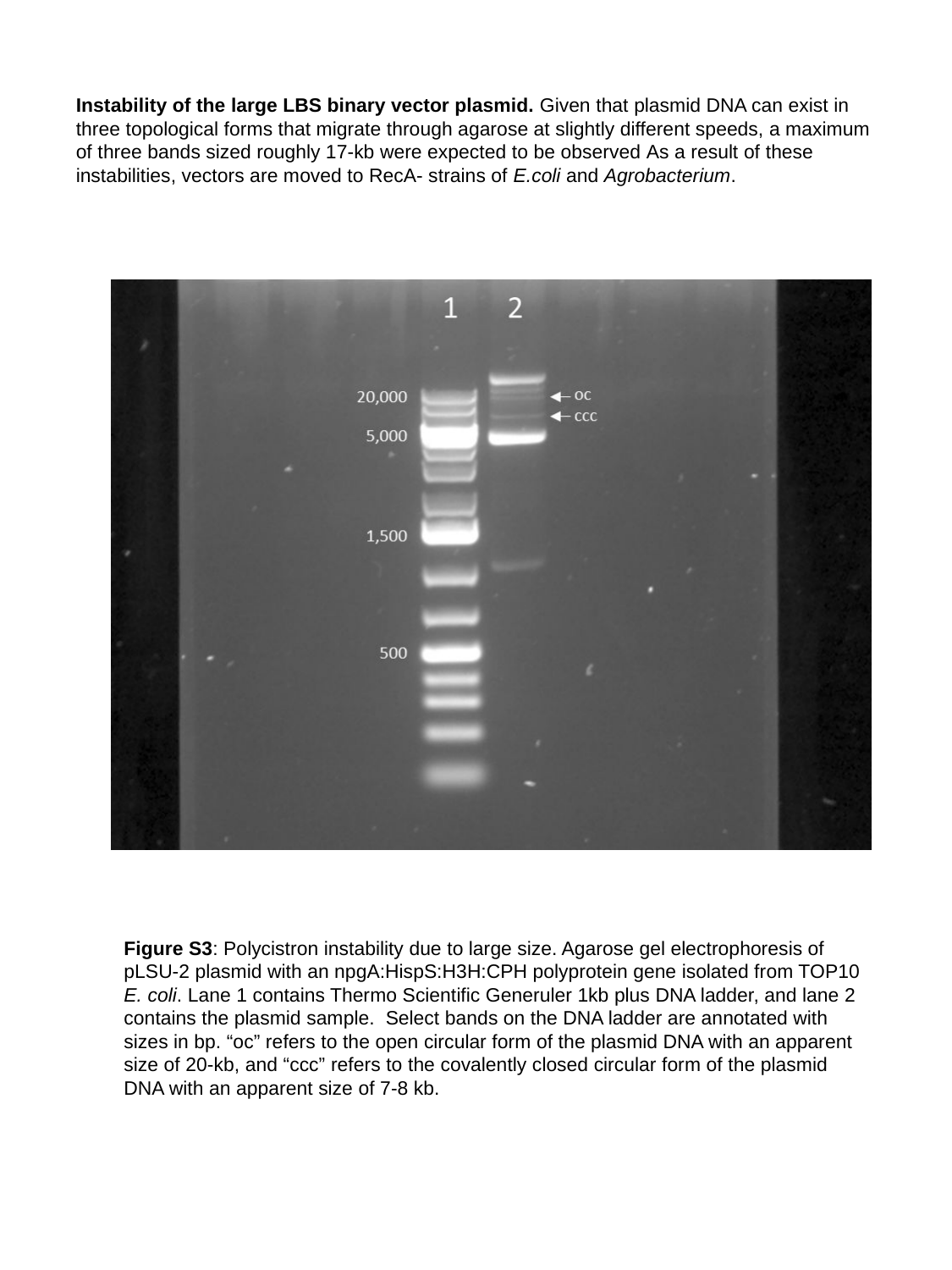

Instability of the large LBS binary vector plasmid. Given that plasmid DNA can exist in three topological forms that migrate through agarose at slightly different speeds, a maximum of three bands sized roughly 17-kb were expected to be observed As a result of these instabilities, vectors are moved to RecA- strains of E.coli and Agrobacterium.
Figure S3: Polycistron instability due to large size. Agarose gel electrophoresis of pLSU-2 plasmid with an npgA:HispS:H3H:CPH polyprotein gene isolated from TOP10 E. coli. Lane 1 contains Thermo Scientific Generuler 1kb plus DNA ladder, and lane 2 contains the plasmid sample. Select bands on the DNA ladder are annotated with sizes in bp. “oc” refers to the open circular form of the plasmid DNA with an apparent size of 20-kb, and “ccc” refers to the covalently closed circular form of the plasmid DNA with an apparent size of 7-8 kb.

## Slide 4
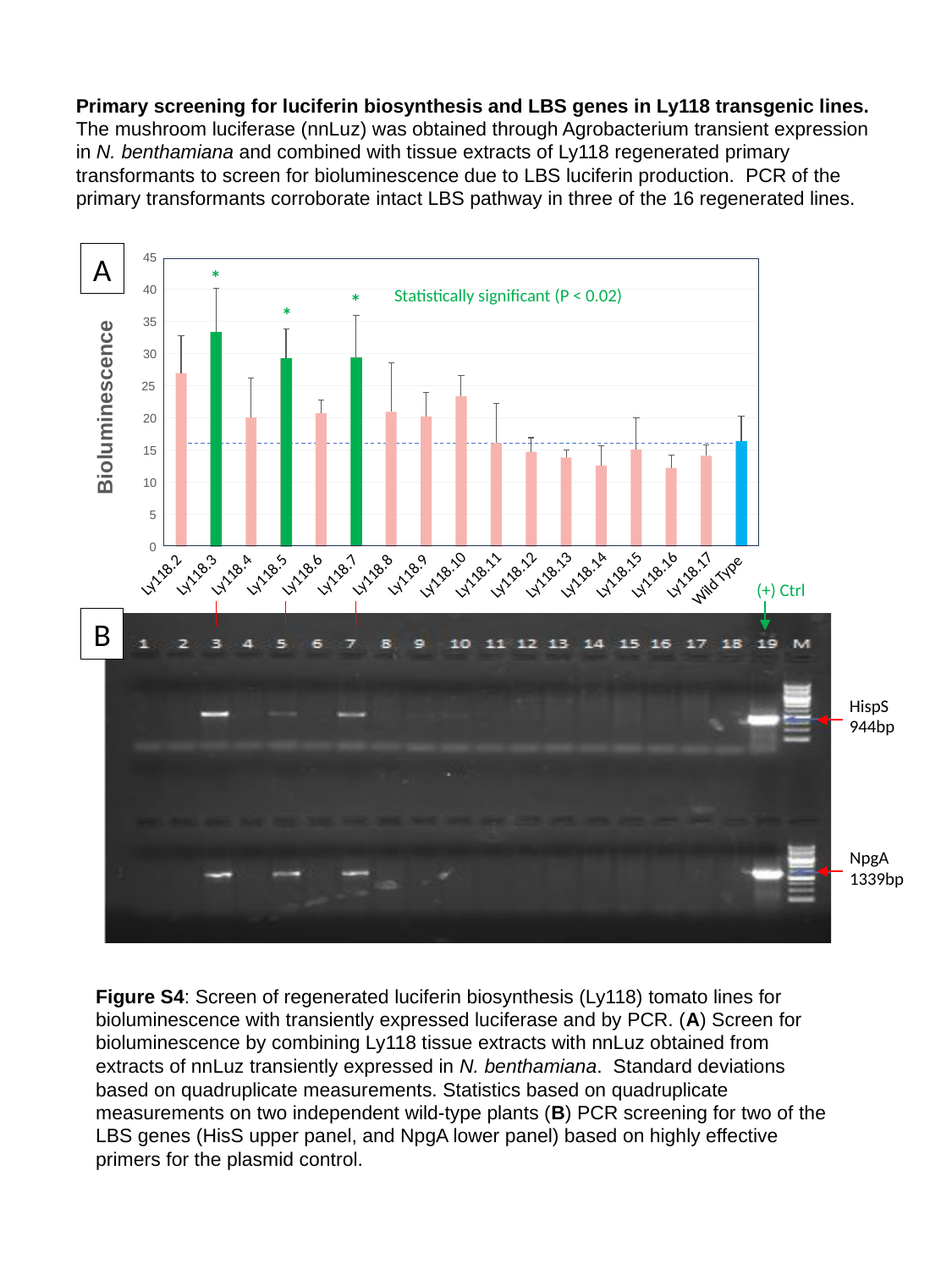

Primary screening for luciferin biosynthesis and LBS genes in Ly118 transgenic lines. The mushroom luciferase (nnLuz) was obtained through Agrobacterium transient expression in N. benthamiana and combined with tissue extracts of Ly118 regenerated primary transformants to screen for bioluminescence due to LBS luciferin production. PCR of the primary transformants corroborate intact LBS pathway in three of the 16 regenerated lines.
A
45
40
35
30
25
20
15
10
5
0
*
Statistically significant (P < 0.02)
*
*
Bioluminescence
Ly118.2
Ly118.3
Ly118.4
Ly118.5
Ly118.6
Ly118.7
Ly118.8
Ly118.9
Ly118.10
Ly118.11
Ly118.12
Ly118.13
Ly118.14
Ly118.15
Ly118.16
Ly118.17
Wild Type
(+) Ctrl
B
HispS
944bp
NpgA
1339bp
Figure S4: Screen of regenerated luciferin biosynthesis (Ly118) tomato lines for bioluminescence with transiently expressed luciferase and by PCR. (A) Screen for bioluminescence by combining Ly118 tissue extracts with nnLuz obtained from extracts of nnLuz transiently expressed in N. benthamiana. Standard deviations based on quadruplicate measurements. Statistics based on quadruplicate measurements on two independent wild-type plants (B) PCR screening for two of the LBS genes (HisS upper panel, and NpgA lower panel) based on highly effective primers for the plasmid control.

## Slide 5
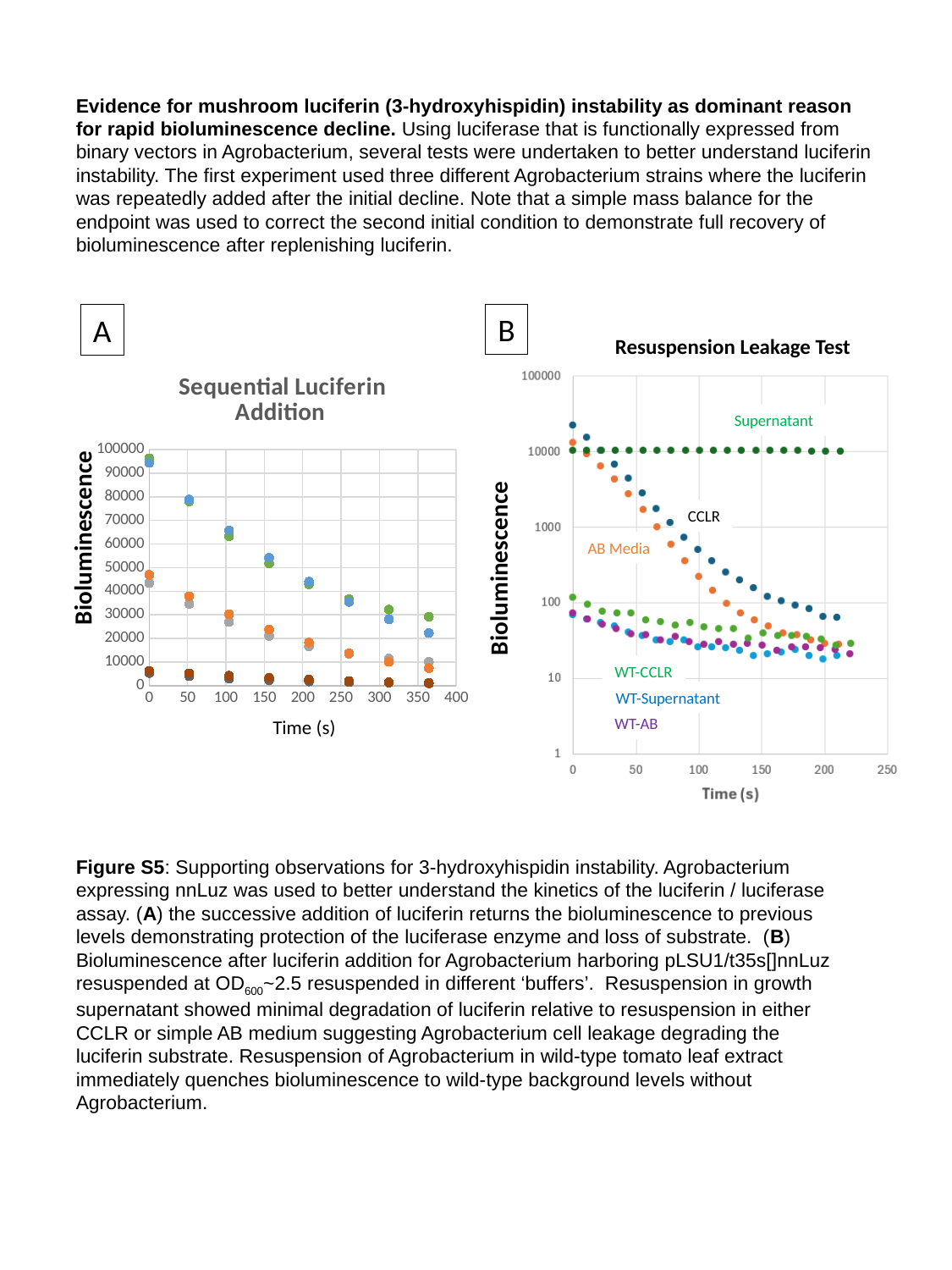

Evidence for mushroom luciferin (3-hydroxyhispidin) instability as dominant reason for rapid bioluminescence decline. Using luciferase that is functionally expressed from binary vectors in Agrobacterium, several tests were undertaken to better understand luciferin instability. The first experiment used three different Agrobacterium strains where the luciferin was repeatedly added after the initial decline. Note that a simple mass balance for the endpoint was used to correct the second initial condition to demonstrate full recovery of bioluminescence after replenishing luciferin.
B
A
Resuspension Leakage Test
### Chart: Sequential Luciferin Addition
| Category | | | | | | |
|---|---|---|---|---|---|---|Supernatant
CCLR
Bioluminescence
AB Media
Bioluminescence
WT-CCLR
WT-Supernatant
WT-AB
Time (s)
Figure S5: Supporting observations for 3-hydroxyhispidin instability. Agrobacterium expressing nnLuz was used to better understand the kinetics of the luciferin / luciferase assay. (A) the successive addition of luciferin returns the bioluminescence to previous levels demonstrating protection of the luciferase enzyme and loss of substrate. (B) Bioluminescence after luciferin addition for Agrobacterium harboring pLSU1/t35s[]nnLuz resuspended at OD600~2.5 resuspended in different ‘buffers’. Resuspension in growth supernatant showed minimal degradation of luciferin relative to resuspension in either CCLR or simple AB medium suggesting Agrobacterium cell leakage degrading the luciferin substrate. Resuspension of Agrobacterium in wild-type tomato leaf extract immediately quenches bioluminescence to wild-type background levels without Agrobacterium.

## Slide 6
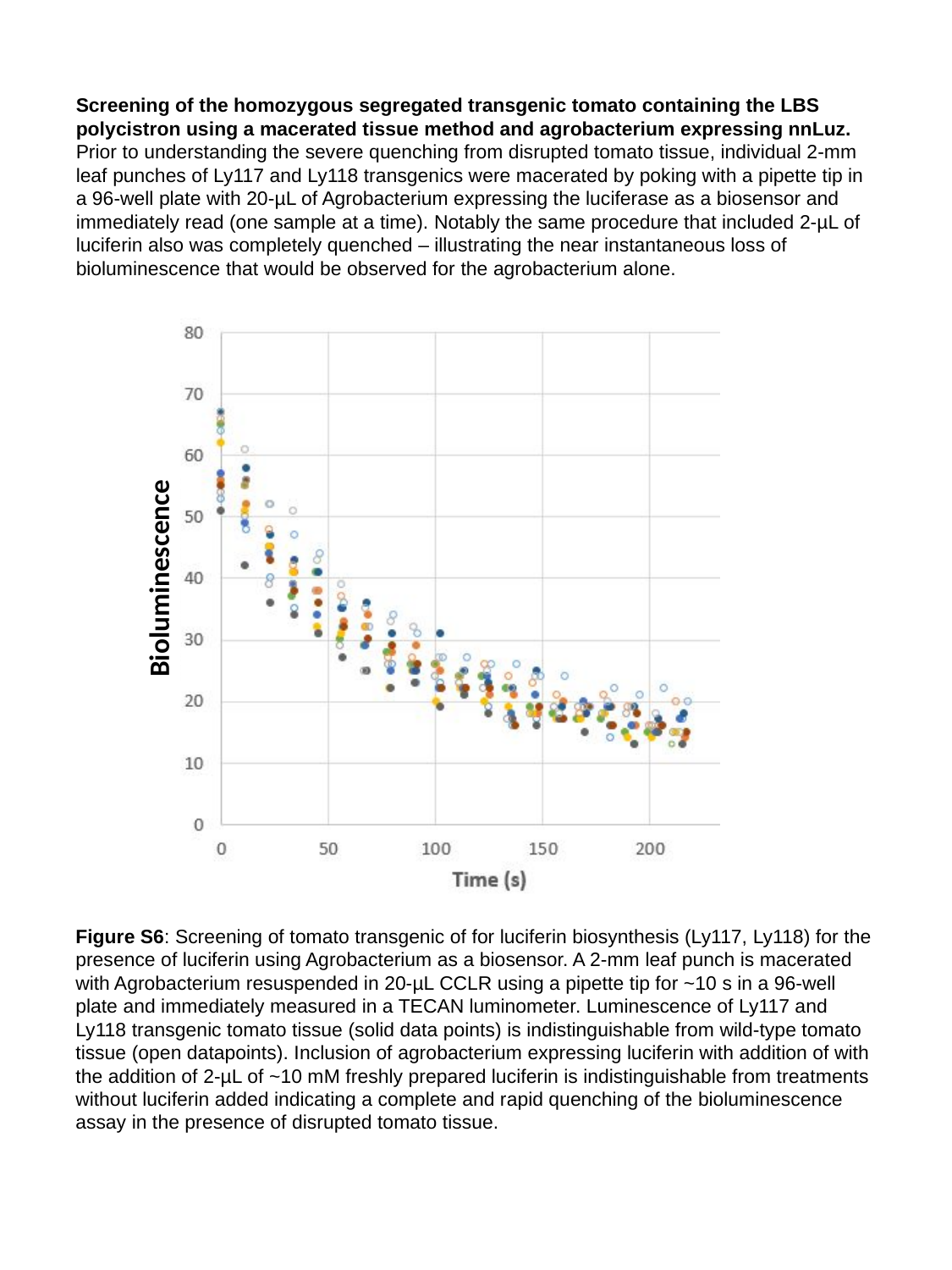

Screening of the homozygous segregated transgenic tomato containing the LBS polycistron using a macerated tissue method and agrobacterium expressing nnLuz. Prior to understanding the severe quenching from disrupted tomato tissue, individual 2-mm leaf punches of Ly117 and Ly118 transgenics were macerated by poking with a pipette tip in a 96-well plate with 20-µL of Agrobacterium expressing the luciferase as a biosensor and immediately read (one sample at a time). Notably the same procedure that included 2-µL of luciferin also was completely quenched – illustrating the near instantaneous loss of bioluminescence that would be observed for the agrobacterium alone.
Bioluminescence
Figure S6: Screening of tomato transgenic of for luciferin biosynthesis (Ly117, Ly118) for the presence of luciferin using Agrobacterium as a biosensor. A 2-mm leaf punch is macerated with Agrobacterium resuspended in 20-µL CCLR using a pipette tip for ~10 s in a 96-well plate and immediately measured in a TECAN luminometer. Luminescence of Ly117 and Ly118 transgenic tomato tissue (solid data points) is indistinguishable from wild-type tomato tissue (open datapoints). Inclusion of agrobacterium expressing luciferin with addition of with the addition of 2-µL of ~10 mM freshly prepared luciferin is indistinguishable from treatments without luciferin added indicating a complete and rapid quenching of the bioluminescence assay in the presence of disrupted tomato tissue.
