# Supplementary material for: Expression of a multigene mushroom luciferin biosynthesis pathway as a pseudo-polycistron in plants
Source: Sci Rep. 2025 Jul 14;15:25385. doi: 10.1038/s41598-025-98717-2 (PMC12259908; doi:10.1038/s41598-025-98717-2)
Supplement: Supplementary file 2 — Supplementary Information 2. [file 41598_2025_98717_MOESM2_ESM.docx]

**Expression of a multigene mushroom luciferin biosynthesis pathway as a pseudo-polycistron in plants**

David Samson, Natalie S. Thompson, Vijay Sheri, Sairam V. Rudrabhatla, Wayne R. Curtis

**Supplemental File 2**

This supplemental document contains additional details for the cloning strategies for the various transient expression and transformation vectors including intron and intein polycistrons. pLSU1 is a compact, high-copy number, and exceptionally stable *Agrobacterium* binary vector that is the template for most of the vectors. Where available, the GenBank and ADDGENE sequences are noted.

**INDEX**

**S2-1: Additional Cloning Details**

**S2-1A: Gibson Assembly and PCR confirmation of LBS polycistrons**

**S2-1B: nnLuz Transient Expression Vectors**

**S2-1C: PCR Primer Table**

**S2-2: Relevant DNA Sequences**

**S2-2A: Luciferase Sequence**

**S2-2B: Intron Location Rationale**

**S2-2C: Non-Redundant intein-F2A Sequences**

**S2-2D: Transposable element disrupting LBS**

**S2-2E: Potato PIV-2 Intron**

**S2-3: Recipes**

**S2-3A: Infiltration Buffer**

**S2-3B: CCLR / AB Media / Phosphate Buffer**

**S2-1: Additional Cloning Details**

**S2-1A: Gibson Assembly and PCR confirmation of LBS polycistrons**

The details and associated schematics are provided as an example of the cloning strategy for the construction of the pLSU-4 Plasmid with a 35S promoter-driven three-enzyme operon for a single transformation of the entire luciferin biosynthesis/recycle pathway for expression in plants.

Overview: The strategy for carrying out this task is to PCR amplify each fragment of the gene with homology to its adjacent fragment conduct a ‘Gibson Assembly’ with these fragments to assemble the gene. This will result in one vector (pLSU-4) with a 35S(0.4-kb):HispS:nrIntF2A-1:H3H:CPH:oIntF2A:Tnos operon in the T-DNA region.

Starting Materials: (Restriction sites in pLSU-4 MCS shown)

pLSU-4

**
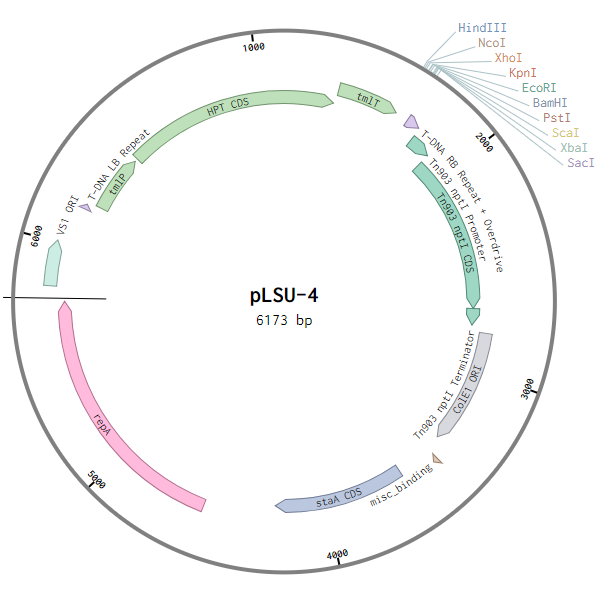
**

0.4-kb 35S Promoter, Modified TMV Ω’ Leader, HispS Coding Sequence (5606bp)

Source: pLSU-2/P35S(0.4-kb)[TMV Ω’]:HispS:Tnos


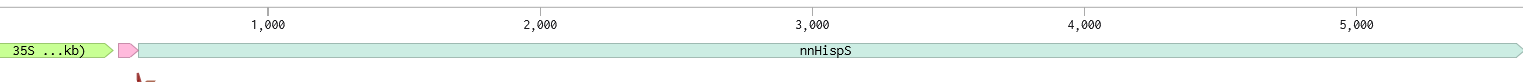


Non-Repetitive Intein/F2A 1 (nrIntF2A-1) Fusion Coding Sequence (675-bp)

Source: Synthesized by IDT, ligated into pJET1.2


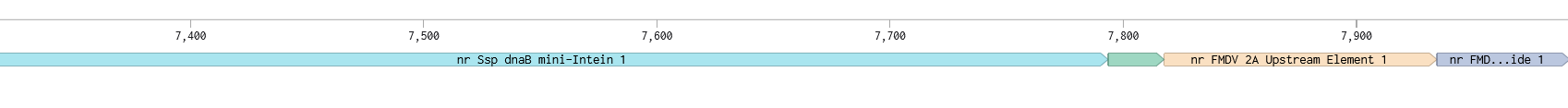


H3H, Original Intein/F2A (oIntF2A), CPH, NOS Terminator (3185-bp)

Source: pLSU-4/P35S(0.4-kb)[TMV Ω’]:H3H:oIntF2A:CPH:Tnos


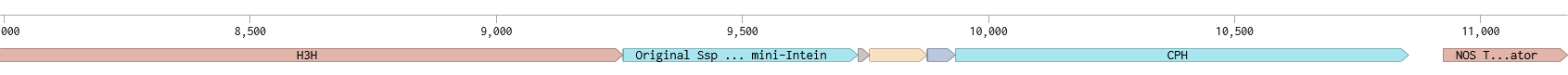


**Details:**

1. PCR amplify (or digest) each fragment
   1. For all fragments in the operon, PCR amplify with homology extensions using Q5 DNA polymerase
      1. For 35S/HispS fragment, use LBS13 (Fwd) and LBS43 (Rev)
      2. For the nrIntF2A-1 fragment, use LBS44 (Fwd) and LBS45 (Rev)
      3. For H3H:oIntF2A:CPH:Tnos fragment, use LBS46 (Fwd) and LBS14 (Rev)
   2. For pLSU-4, digest with KpnI and remove 5’ phosphates with Shrimp Alkaline Phosphatase (to prevent self-ligation during Gibson Assembly)
2. Gibson Assembly of pLSU-4 (KpnI digest, rSAP dephosphorylate), 35S/HispS, nrIntF2A-1, H3H:oIntF2A:CPH: Tnos fragments

**Primers:**

Legend: P35S fragment, HispS, oIntF2A, H3H, CPH, nrIntF2A1, Tnos fragment, pLSU-4

| ID: | Name: | Sequence (5’-3’): |
| --- | --- | --- |
| LBS13 (v2) | pLSU-35S Fwd | ccatggctcgagggtaccGGAGGTCAACATGGTGGAGC |
| LBS43 | Overlap HispS-nrInt1 Rev | TTCAGTACCAAAAGACAGGCAGTTGTCCTCGGAAGCCTC |
| LBS44 | Overlap HispS-nrInt1Fwd | GAGGCTTCCGAGGACAACTGCCTGTCTTTTGGTACTGAA |
| LBS45 | Overlap nrF2A1-H3H Rev | AGTGAATTTTCAAAAGAGGCcatTGGGCCAGGATTGGATTCA |
| LBS46 | Overlap nrF2A1-H3H Fwd | TGAATCCAATCCTGGCCCAatgGCCTCTTTTGAAAATTCACT |
| LBS14 (v2) | Tnos-pLSU Rev | tctgcagggatccgaattcgatctagtaacatagatgacaccg |

**All primers were screened for**

- 1. Matching melting temps of 55^o^C±1^o^C (OligoCalc prediction, nearest neighbor model, 500-nM primer concentration)
  2. No hairpins with Tm within 10^o^C of the primer’s annealing temp (Tm+3 for HF Polymerases) (IDT OligoAnalyzer, 500-nM primer concentration)

1. No self-dimers with ΔG within 20 kcal/mol of the primer-template annealing ΔG (IDT OligoAnalyzer)
2. No hetero-dimers between a possible primer pair with ΔG within 25 kcal/mol (esp. not <20 kcal/mol) of the annealing ΔG of either primer (IDT OligoAnalyzer).
3. No off-target primer-template annealing inside the amplicon with Tm within 10^o^C of the primer’s annealing temp (Benchling)

Example Sequence Confirmation of LBS:

Since the polycistronic constructs were assembled in 2018, full plasmid sequencing was relatively expensive and not readily available. As a result, sequence confirmation was achieved by groups of overlapping PCR segments along these large plasmids. The image below provides an example of such a sequencing effort.


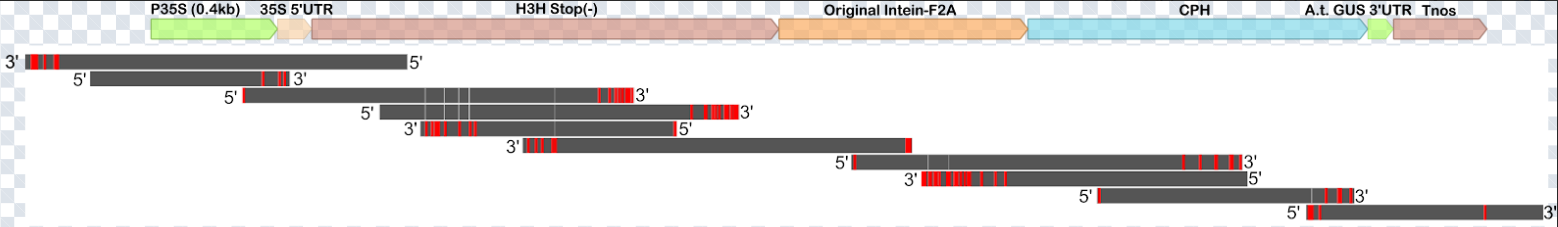


Full plasmid sequencing is now available ‘same day’ for about $8 / plasmid which has allowed for sequence confirmations of the LBS constructs.

**S2-1B: nnLuz Transient Expression Vectors**

Transient expression vectors were created in forms which included the potato PIV intron to prevent expression in Agrobacterium, where those without the intron display expression in Agrobacterium. The truncated cauliflower mosaic virus promoter (t35s) is particularly active and the absence of a 5’UTR typically benefits prokaryotic expression.

***pLSU1/t35s[]:nnLuz:Tnos***. To create pLSU1/t35s[]:nnLuz:Tnos, The mushroom luciferase of *N. nambi* (nnLuz) was amplified by PCR from pX018 with restriction overlap primers {nnLuzIF} and {nnLuzIR}, digested using KpnI and BamHI, and ligated into pLSU1/t35s[]:MCS: Tnos, but with the same enzymes resulting in pLSU1/t35s[]:nnLuz: Tnos. Addgene ID = 212183

***pLSU1/t35s[TMV*** Ω’***]:nnLuz:Tnos***. To create pLSU1/t35s[TMV]:nnLuz:Tnos, The mushroom luciferase of *N. nambi* (nnLuz) was amplified by PCR from pX018 with restriction overlap primers {nnLuzIF} and {nnLuzIR}, digested using KpnI and BamHI, and ligated into pLSU1/t35s[TMV]:MCS:Tnos, resulting in pLSU1/t35s[TMV]:nnLuz:Tnos. Addgene ID = 212188

An analogous procedure was performed with pLSU1/t35s[TMV]:MCS:Tnos, resulting in pLSU1/o35s[TMV]:nnLuz:Tnos. Addgene ID = 212189

***pLSU1/t35s[]: nnLuz-Intron{PIV-216}:Tnos.*** The potato intron-containing versions of the mushroom luciferase nnLuz-I{PIV-216} were constructed using the Gibson Assembly analogous to mNG-I{PIV-216} with pJET1.2/blunt as the destination vector and TOP10 *E. coli* as the cloning strains. The 5’ and 3’ fragments of nnLuz CDS were amplified from pX018 (provided by Planta LLC ahead of Mitiouchkina et al 2020; doi.org/10.1038/s41587-020-0500-9) using overlap primer pairs {nnL5}/{nnL6} and {nnL7}/{nnL8}, respectively. The PIV2 intron was amplified from the pEAQ-GUS plasmid (Larsen and Curtis 2012; doi.org/10.1186/1472-6750-12-21) with overlap primers {nnL9} and {nnL10} as listed in **Table S2-1C** below. For ligation into the pLSU1 drop in expression vector, nnLuz-I{PIV-216} was amplified by PCR from pJET1.2/nnLuz-I{PIV-216} with a 5’ XhoI site extension and a 3’ EcoRI site extension using primers {nnL11} and {nnL12} and then ligated with the pLSU1/t35s:MCS:Tnos backbone and digested with XhoI and EcoRI, to create pLSU1/t35s[]:nnLuz-I{PIV-216}:Tnos. Addgene ID = 212187.

***pLSU1/t35s******[TMV Ω’]:nnLuz-Intron{PIV-216}:Tnos.***

The nnLuz-Intron{PIV-216} was cloned out of pLSU1/t35s:nnLuz-Intron{PIV-216}:Tnos vector with restriction overlap primers {nnLuzIF} and {nnLuzIR}, digested using KpnI and BamHI, and ligated into pLSU1/t35s[TMV]:MCS:Tnos resulting in pLSU1/t35s[TMV]:nnLuz-Intron{PIV-216}:Tnos. Addgene ID = 212186.

**S2-1C: PCR Primer Table**

Relatively complicated cloning such as Gibson Assembly and overlap extension PCR require large numbers of primers. Throughout the text and supplemental there are some primers provided directly where that makes sense, where others are provided as {primer-F/R} to allow for a readable text.

**Table S2-1C – Cloning Primers**

| Primer Name | Sequence |
| --- | --- |
| nnLuzIF | AAAAGGTACCATGAGGATAAATATCTCTTTGTCTA |
| nnLuzIR | AAAAAAGGATCCGAATTCTCAACCTG |
| nnL5 | CTCGAGTTTTTCAGCAAGATATGAGGATAAATATCTCTTTGTCTAG |
| nnL6 | CAAAGGTAGAAGCAGAAACTTACTTGCCTAAAAAGTGACAAGACAC |
| nnL7 | GACCAAAATTTGTTGATGTGCAGGAGCAGAAAGGGTTGGCA |
| nnL8 | ATTATTGTAGGAGATCTTCTAGAAAGATTCAACCTGACTTGGCATTTTC |
| nnL9 | GTGTCTTGTCACTTTTTAGGCAAGTAAGTTTCTGCTTCTACCTTTG |
| nnL10 | TGCCAACCCTTTCTGCTCCTGCACATCAACAAATTTTGGTC |
| nnL11 | AAAACTCGAGATGAGGATAAATATCTCTTTGTCTAG |
| nnL12 | AAAAAGAATTCTCAACCTGACTTGGCATTTTC |
| LBS02 | CATTACAATTACATTTACAATTATCGATACAatgGCCTCTTTTGAAAATTCACTCT |
| LBS03 | AGAGTGAATTTTCAAAAGAGGCcatTGTATCGATAATTGTAAATGTAATTGTAATG |
| LBS04 | CATTACAATTACATTTACAATTATCGATACAatgGCACCTATTTCATCCACGTG |
| LBS05 | CACGTGGATGAAATAGGTGCcatTGTATCGATAATTGTAAATGTAATTGTAATG |
| LBS06 | GAGGCTTCCGAGGACAACTAGatcaacaactctcctggcgc |
| LBS07 | gcgccaggagagttgttgatCTAGTTGTCCTCGGAAGCCTC |
| LBS08 | GGAGCTCTAACTCAGCTTAgatcaacaactctcctggcg |
| LBS09 | cgccaggagagttgttgatcTAAGCTGAGTTAGAGCTCC |
| LBS10 | cgccaggagagttgttgatcTATTCGTGGCCGCCTG |
| LBS11 | CAGGCGGCCACGAATAgatcaacaactctcctggcg |
| LBS13 | ccatggctcgagggtaccGGAGGTCAACATGGTGGAGC |
| LBS14 | tctgcagggatccgaattcgatctagtaacatagatgacaccg |
| LBS35 | CTCAGTTCCGAAGGACAAACAAGCTGAGTTAGAGCTCCTAA |
| LBS36 | TTAGGAGCTCTAACTCAGCTTGTTTGTCCTTCGGAACTGAG |
| LBS37 | CGTGGATGAAATAGGTGCcatGGGCCCAGGGTTGGACT |
| LBS38 | AGTCCAACCCTGGGCCCatgGCACCTATTTCATCCACG |
| LBS43 | TTCAGTACCAAAAGACAGGCAGTTGTCCTCGGAAGCCTC |
| LBS44 | GAGGCTTCCGAGGACAACTGCCTGTCTTTTGGTACTGAA |
| LBS45 | AGTGAATTTTCAAAAGAGGCcatTGGGCCAGGATTGGATTCA |
| LBS46 | TGAATCCAATCCTGGCCCAatgGCCTCTTTTGAAAATTCACT |
| LBS49 | CATTACAATTACATTTACAATTATCGATACAATGGTACAGGATACAAGTTCTGC |
| LBS50 | GCAGAACTTGTATCCTGTACCATTGTATCGATAATTGTAAATGTAATTGTAATG |
| LBS51 | ACAGCGTGACGGCTGAatcaacaactctcctggcg |
| LBS52 | cgccaggagagttgttgatTCAGCCGTCACGCTGT |
| LBS53 | CGACAGCGTGACGGCTGCCTCTCATTTGGGACAG |
| LBS54 | CTGTCCCAAATGAGAGGCAGCCGTCACGCTGTCG |
| LBS55 | AGTCGAACCCAGGGCCTATGAACTCAAGTAAGAATCCGC |
| LBS56 | GCGGATTCTTACTTGAGTTCATAGGCCCTGGGTTCGACT |

**Table S2-1D – Bracket and Sequencing Primers**

| Primer Name | Sequence |
| --- | --- |
| LBS17 | GTATCGATAATTGTAAATGTAATTGTAATG |
| LBS18 | atcaacaactctcctggcgc |
| LBS19 | CCGAGCTCGGCGCAG |
| LBS20 | GCTACGGCGTCCTCCAATC |
| LBS21 | ATGAACTCAAGTAAGAATCCGCCC |
| LBS22 | CAACGCAGTCATGTATAAGGGAG |
| LBS23 | TGGTCACATGTCCTGGTTTTGC |
| LBS24 | CAACAACAGACGATGGTTTTGAAAC |
| LBS25 | GGATTGCTCTAGTGTCCCTTTGTT |
| LBS26 | CTAATGCCCCCGCTGGTATG |
| LBS27 | GATTCTGGTAAGGAGAGTCTCATGG |
| LBS28 | AAATAATCGGAAGCGGCAGTG |
| LBS29 | TGCACTGTATTGGAATATCCTGTG |
| LBS30 | TTCCCCGCATTCCAGGACAT |
| LBS31 | GTGATGGATGGTCCATGTACATCG |
| LBS32 | TCCAACACGATCCCTCCCAG |
| LBS33 | GAGCTACCGGACACAACGGT |
| LBS34 | AAATCGAGGTCCTCGTTGGATTT |
| LBS39 | GAGAACAGGAGGTTCTTGAATATGAG |
| LBS40 | GAGAACAGGAGGTTCTTGAATATGAG |
| LBS41 | GAGGAGCATCGTGGAAAAAG |
| LBS42 | CCATATGCTTCACGGTAATCA |
| LBS57 | GTGAATGAAAGGCAAGGGC |
| LBS58 | TGCCTCTCATTTGGGACAG |
| pLSU2-4 5' Fwd | TTCTAAAAGTGTTCTAAGCGGGC |
| pLSU-1 3' R | tgtattactgtttatgtaagcagaca |

**S2-2: Relevant DNA Sequences**

**S2-2A: Luciferase Sequence**

*Mushroom luciferase (nnLuz)*

ATGAGGATAAATATCTCTTTGTCTAGTCTCTTTGAGAGACTGAGCAAATTGTCATCCCGATCCATCGCAATTACCTGCGGCGTAGTCTTGGCAAGTGCAATAGCATTCCCAATTATCCGAAGAGACTATCAAACGTTCCTTGAAGTCGGTCCGAGCTATGCCCCACAGAACTTCCGAGGCTATATCATCGTTTGTGTCTTGTCACTTTTTAGGCAAGAGCAGAAAGGGTTGGCAATCTATGACAGGCTTCCAGAAAAGAGGCGTTGGCTTGCCGACTTGCCGTTTCGTGAAGGGACGAGGCCATCCATAACCTCCCACATTATACAGCGTCAGCGTACTCAGCTGGTAGATCAAGAGTTTGCAACCAGAGAACTTATCGATAAGGTGATCCCACGTGTGCAAGCAAGACACACAGACAAAACTTTTCTCAGTACGTCAAAATTTGAGTTTCATGCAAAGGCCATCTTCCTTCTCCCCTCTATCCCTATTAATGATCCTCTTAACATTCCCTCACACGATACGGTAAGAAGAACCAAGCGTGAGATCGCTCACATGCATGATTACCATGATTGCACTCTGCACTTGGCTCTTGCTGCTCAGGATGGTAAGGAAGTTTTGAAGAAGGGGTGGGGCCAGCGTCACCCACTGGCCGGACCAGGAGTCCCAGGCCCTCCTACTGAGTGGACCTTCCTTTACGCACCAAGGAACGAAGAGGAGGCCAGAGTTGTCGAGATGATAGTCGAAGCTAGTATTGGCTACATGACTAACGATCCTGCTGGTAAGATTGTCGAAAATGCCAAGTCAGGTTGA

**S2-2B: Intron Location Rationale**

Since no structure is available, the choice of intron insertion for this luciferase was based on examining the conserved sequence regions and structure prediction from only software.


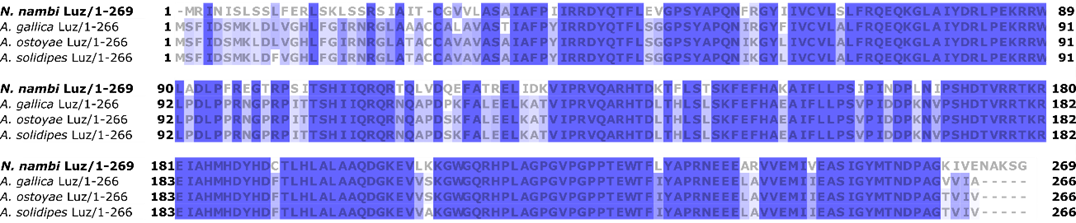


**Figure** **S2-2B.1:**  Multiple sequence alignment of amino acid sequences of *N. nambi* Luz homologs obtained by tBLASTn for which there are known bioluminescent mushrooms. Conserved regions are highlighted in dark blue.


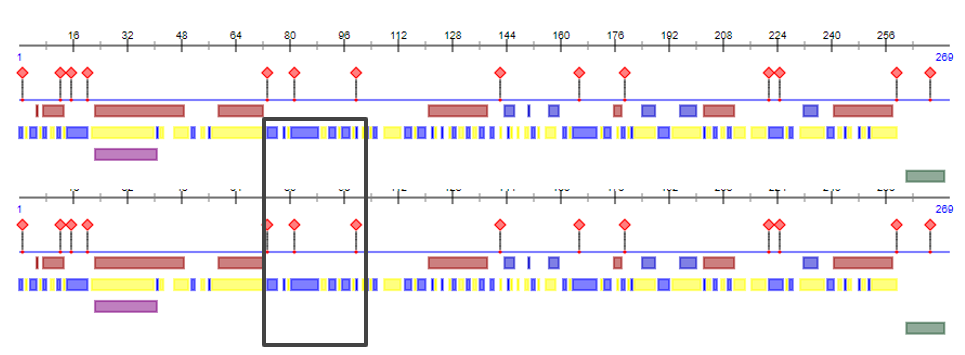


**Figure S2-2B.2:** Secondary structure prediction of nnLuz. The amino acid sequence of nnLuz was modeled in PredictProtein (predictprotein.org/, ver. July 2013), which predicts protein secondary structures, solvent accessibility, and protein and nucleic acid binding sites within the protein. The conserved region of L71-L92 consisted of part of a predicted completely buried alpha-helix and a region of mostly solvent-exposed residues (which also makes a case for it being part of the enzyme’s active site). In addition, this region and the region downstream of it (L71-Q117) were predicted to not have any canonical secondary structure. Finally, the protein did not have any predicted disulfide bridges.

-Alpha Helix -Buried Residues -Solvent-Exposed Residues -Protein Binding Site

-Transmembrane Helix -Disordered Region


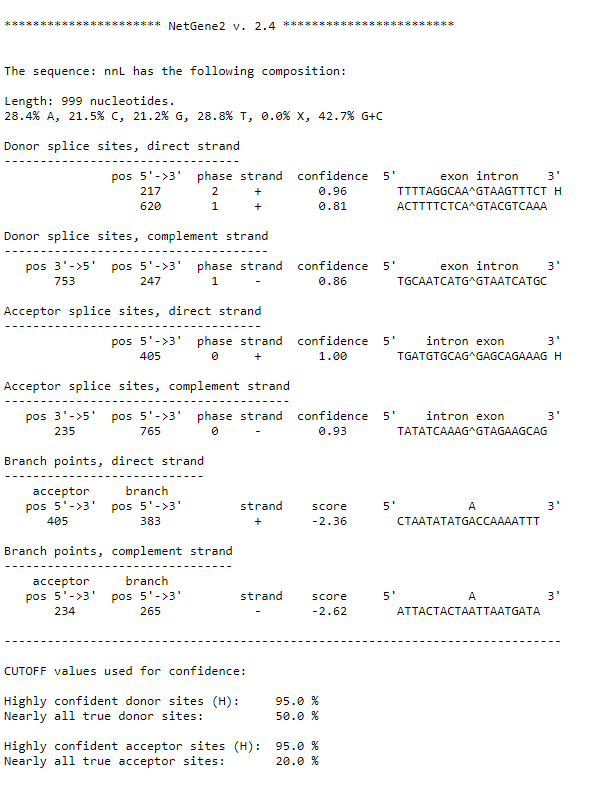


**Figure S2-2B.3: Intron location approach for Reporter Genes.** Candidate nnLuz-Intron CDS in NetGene2 splice site prediction tool (cbs.dtu.dk/services/NetGene2/ ; ver. 2.42 (Hebsgaard et al. 1996)) with settings for *A. thaliana* to see if it predicted that it contained a donor, acceptor and branch point site corresponding to the inserted intron.

**S2-2C: Non-Redundant intein-F2A Sequences**

The F2A inteins facilitate ribosome skipping to re-initiate translation after the 3’ NPGP (underlined) sequence https://doi.org/10.1508/cytologia.84.107. All inteins code for the same amino acid sequence: MVKVIGRRSLGVQRIFDIGLPQDHNFLLANGAIAAACSCGSGSRVTELLYRMKRAETYCPRPLLAIHPTEARHKQKIVAPVKQLLNFDLLKLAGDVESNPGP

*Non-homologous Intein Sequences:*


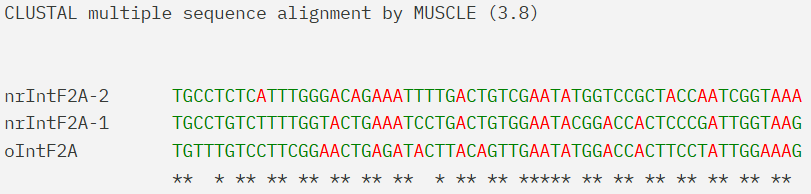


**nrIntF2A-1**

TGCCTGTCTTTTGGTACTGAAATCCTGACTGTGGAATACGGACCACTCCCGATTGGTAAGATTGTATCTGAAGAAATAAATTGCTCTGTTTACTCTGTTGATCCAGAAGGGCGGGTGTATACCCAAGCCATTGCTCAATGGCATGACCGTGGTGAGCAGGAAGTACTCGAATACGAGTTAGAGGATGGTAGTGTAATTCGGGCCACCTCCGATCATAGGTTCCTGACAACAGACTACCAGCTCCTTGCCATAGAAGAAATATTCGCGCGGCAACTAGATCTCCTGACCTTGGAAAATATCAAGCAAACAGAGGAAGCGCTTGACAACCACCGACTGCCGTTTCCACTATTAGATGCTGGTACAATCAAGATGGTAAAGGTAATCGGGCGCCGAAGTTTGGGGGTACAAAGGATATTCGACATTGGGCTTCCTCAAGATCATAATTTTCTATTAGCGAATGGTGCCATAGCCGCCGCGTGCAGCTGTGGGTCGGGCTCTAGGGTAACCGAGTTGCTCTACCGCATGAAACGCGCTGAAACTTACTGCCCTCGACCACTACTGGCTATTCATCCGACTGAAGCTCGCCACAAGCAGAAAATAGTAGCACCCGTGAAACAATTACTCAACTTTGACCTATTGAAGTTAGCTGGTGATGTTGAATCCAATCCTGGC**CCA**

**nrIntF2A-2**

TGCCTCTCATTTGGGACAGAAATTTTGACTGTCGAATATGGTCCGCTACCAATCGGTAAAATCGTATCGGAAGAAATCAATTGCAGCGTGTACAGTGTCGATCCTGAAGGCCGAGTTTACACGCAGGCGATTGCACAATGGCATGATCGTGGAGAACAGGAAGTATTAGAGTATGAGCTCGAAGACGGGTCAGTAATCCGTGCGACCAGTGACCATAGATTCCTAACAACTGACTATCAATTACTCGCCATTGAAGAAATATTTGCTCGACAATTAGACCTGTTAACGCTGGAAAACATTAAGCAGACGGAAGAAGCACTCGACAATCACAGGTTGCCTTTCCCCCTCCTTGACGCAGGAACGATTAAGATGGTCAAGGTTATCGGGAGAAGGTCGCTGGGTGTCCAGAGGATTTTCGACATCGGCTTACCCCAAGATCACAACTTCCTCCTCGCAAACGGAGCTATCGCTGCTGCATGCTCTTGTGGTTCCGGTTCCCGTGTAACGGAGCTCTTGTACCGGATGAAGAGAGCCGAAACTTATTGTCCACGACCTTTGTTGGCGATCCACCCGACTGAGGCTCGGCATAAACAGAAGATAGTAGCCCCCGTAAAGCAGCTGCTAAATTTTGATCTCCTCAAACTGGCTGGGGATGTCGAGTCGAACCCAGGG**CCT**

**oIntF2A**

TGTTTGTCCTTCGGAACTGAGATACTTACAGTTGAATATGGACCACTTCCTATTGGAAAGATTGTGAGTGAAGAGATCAACTGCAGTGTTTATTCCGTGGATCCAGAGGGTAGAGTTTACACTCAAGCAATTGCTCAGTGGCATGATAGGGGAGAACAGGAGGTTCTTGAATATGAGTTGGAAGATGGTTCTGTGATAAGAGCTACATCAGATCACAGGTTTCTTACTACAGATTACCAACTTTTGGCAATCGAAGAGATTTTCGCTAGACAGCTCGATCTTCTCACTTTGGAAAATATTAAGCAAACAGAAGAGGCACTTGATAACCATAGGCTTCCATTTCCTCTTTTGGATGCTGGAACTATTAAGATGGTTAAAGTGATAGGAAGAAGGTCATTGGGTGTTCAAAGAATATTTGATATCGGACTTCCTCAGGATCACAATTTCTTACTCGCAAACGGTGCTATTGCTGCAGCTTGTTCTTGTGGTTCTGGTTCTAGAGTTACTGAGCTTTTGTATAGGATGAAGAGGGCAGAAACATACTGCCCAAGACCTTTACTCGCAATCCATCCAACAGAGGCTAGGCACAAGCAAAAAATTGTTGCTCCTGTGAAACAGCTTTTGAACTTTGATCTTCTCAAGCTTGCGGGAGACGTCGAGTCCAACCCTGGG**CCC**

**S2-2D: Transposable element disrupting LBS**

An Agrobacterium isolate containing the LBS pathway lost the ability to confer bioluminescence, while also displaying a recovery of growth rate. It was speculated that this was caused by disruption of the LBS polycistron, thereby alleviating a cellular toxicity from the LBS transgenes. A plasmid prep was generated from the Agrobacterium strain containing the defective pLUS4//HisS:H3H:CPH 3xLBS and submitted to the Penn State Core facility for whole plasmid sequencing. The results reveal a 1324-bp insertion 3361-bp into the HispS gene (5097-bp total). The insertion is homologous to a IS3 family transposase IS426. The coding sequence is homologous with sequences in the cys32 genome.

>GADEOJFP_04342 IS3 family **transposase IS426**

MKAVADTLGVSRSNLIERLKGRSKPRGPYNKAEDAELLPAIRRLVDQRPTYGYRRIAALLNRERRAADQPVVNAKRVHRIMGNHAMLLE**K**HTAVRKGRLHDGKVMVMRSNLRWCSDGLEFACWNGEVIRLAFIIDAFDREIIAWTAVANAGISGSDVRDMMLEAVEKRFHATRAPHAIEHLSDNGSAYTARDTRLFAQALNLTPCFTPVASPQSNGMSEAFVKTLKRDYIRISALPDAQTALRLIDGWIEDYNEIHPHSALKMASPRQFIRAKSI

The complete sequence of the transposon

ACAAGTGGAGTGCACCCCATTTCACCGGACAAGTCGGCTAGATTGATTTAGCCCTGATGAACTGCCGAGGGGAAGCCATCTTGAGCGCGGAATGGGGATGGATTTCGTTGTAGTCCTCGATCCATCCGTCGATGAGCCGGAGCGCTGTTTGGGCGTCCGGTAGAGCTGATATCCGAATATAGTCCCGCTTCAACGTTTTGACGAAGGCTTCCGACATGCCGTTCGACTGCGGGCTGGCGACCGGCGTGAAGCAGGGCGTGAGATTGAGTGCTTGCGCAAACAGCCTCGTGTCCCGCGCGGTATAAGCCGAGCCATTGTCAGAGAGATGCTCGATAGCATGCGGGGCTCGGGTTGCATGGAAGCGTTTCTCGACCGCCTCCAACATCATGTCGCGCACGTCTGAGCCGGAAATGCCTGCATTGGCAACGGCCGTCCAGGCGATGATCTCGCGGTCGAAGGCGTCGATGATGAAGGCGAGACGAATGACCTCGCCATTCCAGCAGGCGAACTCCAGGCCGTCCGAGCACCAGCGCAGGTTGGAGCGCATGACCATGACCTTGCCATCGTGGAGGCGGCCCTTGCGAACGGCTGTGTGCTTCTCCAGTAGCATGGCGTGGTTACCCATGATGCGATGGACCCGTTTGGCGTTGACGACAGGCTGATCGGCGGCTCGCCTTTCGCGATTGAGGAGCGCGGCGATCCGCCGATAGCCATAGGTTGGCCTTTGATCCACCAGCCTGCGGATGGCGGGCAGAAGCTCTGCATCCTCGGCCTTGTTGTATGGCCCACGCGGCTTTGATCTGCCTTTCAGCCGCTCGATGAGGTTGGAACGGGAGACGCCCAGCGTGTCTGCGACGGCCT**TCAT**CGCGAACCGTCCTTCGGCAACAAGATCGGCCGCGATATCCGTTTTTTTGAGTCCGCTTTGGAAAGGGCTTCGCGGAGGATTTCGACCTCCATCGTCTTGCGACCGAGCATGCGCTCCAACTCCCGGACGCGATCCTCCAGTTTCTTCACTTCCGAATTCCCGACAACCGGCTCGTCAGAATCCACGGCTGCAGCACCTCCCTCGCTCAAGAGCCTGCGCCACCGATAAAGCAAATTGGGCGCCACGCCATGACGGCGAGCGGTCGAAGATACCGTCTCGCCGGGTTCAAAACTCTGCTCAATGATTGTCAGCTTTTGCTCGGTTGTCCACCGCCTGCGGCGAACATCACCCGTCAGCAATTCAACGTGTCGATAGTCGTTAGACATAAGCCTGTCCTCAAGCCTGTGCTTGAGCCTTTCTGCTTATGCCGACTGTCCGGTCGAAATGGGGGGCAGTTCA

**S2-2E: Potato PIV-2 Intron**

*Potato, Solanum tuberosum (PIV, 189-bp) – underlined are minor variations of GenBank ID X04753.1*

gt**aa**gtttctgcttctacctttgatatatatataataattatcattaattagtagtaatataatatttcaaatatttttttcaaaataaaagaatgtagtatatagcaattgcttttctgtagtttataagtgtgtatattttaatttataacttttctaatatatgaccaaaa**tt**tg**t**tgatgt**gc**ag

**S2-3: Recipes**

**S2-3A: Infiltration Buffer**

Agrobacterium Transient Infiltration Buffer Recipe: 10 mM MES. 10 mM MgCl_2_

Stock solutions used to prepare infiltration Buffer

• 1-M MES (100-mL) pH 5.2

To make 100 mL of 1-M stock: Dissolve 21.32-g MES (FW 213.2) in 80-mL milliQ H_2_O, stir and heat (65 ^o^C), to dissolve adjust pH with 10-N NaOH (approx. 600-µL), then bring the volume up to 100-mL with milliQ H_2_O and autoclave for 15 min. at 121°C.

• 1-M MgCl_2_

Dissolve 20.33-g (FW 203.3) in 100-mL of milliQ H_2_O. Stir until completely dissolved and autoclave for 15 min. at 121 °C.

**S2-3B: CCLR / AB Media / Phosphate Buffer**

*CCLR; (a Promega product):*

Used for protein extraction when it is important to maintain enzymatic activity, such as when a luciferase assay is being performed. This is used routinely in the lab for the nanoLuciferase assay. CCLR was also initially used to resuspend the 3-hydroxyhispidin DMSO stock (before switched to the use of small DMSO cryo-aliquots resuspended in phosphate buffer). Agrobacterium was resuspended in CCLR in the study of leakiness that caused rapid luciferin decay.

(https://www.promega.com/products/luciferase-assays/reporter-assays/luciferase-cell-culture-lysis-reagent/?catNum=E1531).

| **Material** | **Stock concentration** | **Final Concentration** | **Amount added [50 mL]** | **Amount added [5 mL]** |
| --- | --- | --- | --- | --- |
| Tris-phosphate (pH 7.8) | 1 M | 25 mM | 1.25 mL | 125 µL |
| DTT | 1 M | 2 mM | 100 µL | 10 µL |
| 1,2 - diaminocyclohexane-N,N,N’,N’-tetraacetic acid | 1 M | 2 mM | 100 µL | 10 µL |
| Glycerol | 60 % | 10 % | 8.33 mL | 833 µL |
| Triton X-100 | 100 % | 1 % | 500 µL | 50 µL |
| Make to final volume with sterile RODI H_2_O | | | |  |
